# The Mutational Landscape of the Oncogenic MZF1 SCAN Domain in Cancer

**DOI:** 10.3389/fmolb.2016.00078

**Published:** 2016-12-15

**Authors:** Mads Nygaard, Thilde Terkelsen, André Vidas Olsen, Valentina Sora, Juan Salamanca Viloria, Fabio Rizza, Sanne Bergstrand-Poulsen, Miriam Di Marco, Mette Vistesen, Matteo Tiberti, Matteo Lambrughi, Marja Jäättelä, Tuula Kallunki, Elena Papaleo

**Affiliations:** ^1^Computational Biology Laboratory and Center for Autophagy, Recycling and Disease, Danish Cancer Society Research CenterCopenhagen, Denmark; ^2^Department of Biomedical Sciences, University of PaduaPadua, Italy; ^3^Cell Stress and Survival Unit and Center for Autophagy, Recycling and Disease, Danish Cancer Society Research CenterCopenhagen, Denmark; ^4^Department of Chemistry and Biochemistry, School of Biological and Chemical Sciences, Queen Mary University of LondonLondon, UK; ^5^Unit of Cell Death and Metabolism and Center for Autophagy, Recycling and Disease, Danish Cancer Society Research CenterCopenhagen, Denmark

**Keywords:** transcription factors, molecular dynamics, protein structure network, TCGA, cancer mutations, FoldX, saturation mutagenesis, RNAseq

## Abstract

SCAN domains in zinc-finger transcription factors are crucial mediators of protein-protein interactions. Up to 240 SCAN-domain encoding genes have been identified throughout the human genome. These include cancer-related genes, such as the myeloid zinc finger 1 (*MZF1*), an oncogenic transcription factor involved in the progression of many solid cancers. The mechanisms by which SCAN homo- and heterodimers assemble and how they alter the transcriptional activity of zinc-finger transcription factors in cancer and other diseases remain to be investigated. Here, we provide the first description of the conformational ensemble of the MZF1 SCAN domain cross-validated against NMR experimental data, which are probes of structure and dynamics on different timescales. We investigated the protein-protein interaction network of MZF1 and how it is perturbed in different cancer types by the analyses of high-throughput proteomics and RNASeq data. Collectively, we integrated many computational approaches, ranging from simple empirical energy functions to all-atom microsecond molecular dynamics simulations and network analyses to unravel the effects of cancer-related substitutions in relation to MZF1 structure and interactions.

## Introduction

Transcription factors belonging to the SCAN zinc finger family have been implicated in a number of cellular malignancies (Monaco et al., 1998; Dong et al., 2004; Yang et al., 2011; Eguchi et al., 2015; Singh et al., 2015). SCAN domains in zinc finger transcription factors are crucial mediators of protein-protein interactions (Williams et al., 1995; Sander et al., 2003; Edelstein and Collins, 2005; Noll et al., 2008) and allow SCAN zinc finger proteins to form homo- and hetero-dimers (Edelstein and Collins, 2005). The importance of dimerization for SCAN zinc finger transcriptional activity was demonstrated through two-hybrid experiments, using ZNF174. This study demonstrated that interaction between SCAN domains synergistically activated transcription (Williams et al., 1999).

SCAN domains have been identified in more than 80 zinc finger genes throughout the human genome (http://www.ebi.ac.uk/interpro/entry/IPR003309/proteins-matched?species=9606), including a number of cancer-related genes. In addition, a subset of SCAN domain only factors (SCAND), which lack the DNA binding domains, has been discovered and suggested to function as regulators of the intact SCAN zinc finger factors (Sander et al., 2003; Edelstein and Collins, 2005).

SCAN domain is a highly conserved domain of ~80 residues and it is usually located in the N-terminal region of transcription factors (Sander et al., 2003). It is composed by at least three α-helices separated by short loop regions. SCAN domains have an extended conformation, also defined as V-shaped structure, composed by five α-helices arranged in two subdomains. The N-terminal subdomain comprises the α-helices 1 and 2 that form one side of the V-shape, whereas the C-terminal subdomain is composed by the α-helices 3, 4, and 5 that pack together forming the other half of the V-shape. In some SCAN domains, the N-terminal subdomain of one monomer packs with the C-terminal subdomain of the other monomers so the two monomers interact in a domain-swapped topology (Peterson et al., 2006).

One example of a SCAN zinc finger transcription factor with a crucial role in cancer is Myeloid Zinc Finger 1 (MZF1). There are three known isoforms of *MZF1* which may play different roles in tumorigenesis—these encode protein sequences of different lengths and domain composition (Peterson and Morris, 2000; Eguchi et al., 2015). The shortest *MZF1* isoform encodes a 290-residue protein, including the SCAN domain, a portion of the regulatory linker and a unique C-terminal motif (Eguchi et al., 2015).

The existence of highly conserved SCAN domains in the SCAN zinc finger family suggests that interactions between its family members may occur through hetero-dimerization (Edelstein and Collins, 2005). MZF1 has been shown to interact with its family members such as ZNF24, ZNF174, and ZNF202 through SCAN-SCAN interactions (Noll et al., 2008) along with SCAND proteins such as RAZ1 or SCAND1/RAZ108 (Sander et al., 2000). The role of the SCAN-SCAND interaction is still unclear, however in the case of MZF1, it has been suggested that the “zinc fingerless” SCAND proteins might decrease MZF1 signaling causing a decrease in the affinity for DNA targets or other interactions (Sander et al., 2000). The N-terminal region of MZF1, which includes the SCAN domain, can also co-associate with the promyelocytic leukemia nuclear bodies (PML-NB, Bernardi and Pandolfi, 2007; Noll et al., 2008) and recruit other factors, such as ZNF24, to the PML-NBs (Noll et al., 2008).

MZF1 was initially associated with malignancies in studies on hematopoietic development, as it was found to act as a transcriptional repressor of essential genes for hematopoietic differentiation. In addition, MZF1 promotes the emergence of a leukemic phenotype (Perrotti et al., 1995; Hromas et al., 1996). On the contrary, Gaboli and co-worker suggested that MZF1 could have a suppressor function on hematopoietic cancer types (Gaboli et al., 2001). The source of these discrepancies might be related to the existence of the different *MZF1* transcripts, which may have diverse functions in the cell (Peterson and Morris, 2000).

The *cBioPortal* database (Cerami et al., 2012) reports the *MZF1* gene to be significantly amplified in certain solid tumors, such as breast, uterine, lung, and bladder cancers (Eguchi et al., 2015). A complex and heterogeneous role of MZF1 in tumors is supported by studies in cellular and animal models of different types of cancer (Hsieh et al., 2007; Mudduluru et al., 2010; Rafn et al., 2012; Chen et al., 2014; Tsai et al., 2015; Vishwamitra et al., 2015; Nan et al., 2016). The effects mediated by MZF1 seem to be strictly dependent on the cancer type. Thus, the interactions and functions involving this transcription factor will require further elucidation on a detailed structural level. Indeed, this is a critical step for the understanding of the molecular mechanisms involved in cancer initiation and development, as well as in the design of new therapeutic approaches.

Despite the many questions surrounding MZF1 and its role in cancer, no systematic studies have been devoted to understanding the structural and functional impact of cancer-related mutations involving this SCAN domain. A NMR structure of the MZF1 SCAN domain [Protein Data Bank (PDB) entry: 2FI2; (Peterson et al., 2006)] in its dimeric form is publicly available and we here use it to study the cancer-related mutational landscape of MZF1.

We analyzed the network of experimentally characterized protein-protein interactions of MZF1 and predicted the candidates for SCAN-SCAN or SCAN-SCAND interactions using an *in silico* high-throughput approach based on the *PRISM* energy function (Tuncbag et al., 2011; Baspinar et al., 2014). The expression levels of MZF1 and its protein partners were compared in normal and tumor samples using RNASeq data from cancer patients. In particular, we applied a Pan Cancer approach to 24 different RNASeq data sets obtained from *The Cancer Genome Atlas* (Tomczak et al., 2015).

We also integrated different computational techniques, such as sequence- and structure-based methods to predict the effects of mutations on protein stability and protein-protein interactions (Schymkowitz et al., 2005), microsecond atomistic molecular dynamics (MD) simulations and methods inspired by graph theory applied to MD (Invernizzi et al., 2014; Tiberti et al., 2014; Papaleo, 2015; Papaleo et al., 2016). In addition, we used high quality NMR data, such as Nuclear Overhauser Effect (NOE) experiments and chemical shifts (BMRB accession number: 6957, Peterson et al., 2006) to cross-validate our MD structural ensembles and compare the results achieved by the different MD force fields.

## Materials and methods

### Protein-protein interactions of MZF1 SCAN domain

We used the *Interologous Interaction Database* (*I2D*; Kotlyar et al., 2016), downloaded on January 8th 2016, to retrieve known interaction partners of human MZF1 (Uniprot identifier P28698). Gene ontology enrichment was performed using the *gprofiler* (Reimand et al., 2007), *GOsim* (Fröhlich et al., 2007), and *corrplot* (Friendly, 2002) packages for R. The interaction network was filtered to remove redundant edges and visualized using *Cytoscape* version 3.3.0 (Shannon et al., 2003). For each MZF1 partner sequence, we performed a search for SCAN and zinc finger domains using *InterPro* (Mitchell et al., 2014) and *Pfam* (Finn et al., 2016).

We then used *PRISM* (Tuncbag et al., 2011; Baspinar et al., 2014) which is a template-based software to predict the structure of protein-protein complexes. *PRISM* includes a rigid-body structural comparison of target proteins to known templates of protein-protein interfaces and a flexible refinement using a docking energy function. We used the standalone version of the program to screen all the interactors collected by high-throughput experimental studies for their interaction with the SCAN domain of MZF1. The interaction partners selected in the first *PRISM* round were then submitted to *PRISM 2.0* and evaluated on the basis of docking energy scores using as a cutoff for favorable interactions a docking energy value of −2.39 kcal/mol, as previously suggested (Baspinar et al., 2014; Tuncbag et al., 2011).

The flexible refinement in PRISM was performed using Fiberdock (Mashiach et al., 2010) including a step to remove steric clashes. The resulting complexes are then ranked by binding energy scores (i.e., Docking energy) calculated using the CHARMM force field (MacKerell et al., 1998).

### Analyses of the Cancer Genome Atlas (TCGA) RNASeq data

Level 3 RNASeq data (RSEM counts) for all the cancer studies deposited at *The Cancer Genome Atlas (TCGA)* were downloaded and pre-processed using the R-package *TCGAbiolinks* (Colaprico et al., 2015; Silva et al., 2016). The RNASeq data used for analyses had been produced using the Illumina HiSeq 2000 mRNA sequencing platform. For the analysis, we retained only those data sets for which both tumor and normal samples were available. A summary of the analyzed samples and cancer types is reported in Table S1.

Before the analyses, sample outliers were removed using the *TCGAAnalyse_Preprocessing* function from *TCGAbiolinks*, which is a function that estimates the Pearson correlation coefficient among all pairs of samples (38). Samples with a correlation lower than 0.6 within the same cancer study were discarded (38). Next, we normalized the expression data using the *TCGAnalyze_Normalization* function from *TCGAbiolinks*. Normalization procedures included adjusting for GC-content, gene-length effects on read counts and full quantile filtering of datasets using a cutoff value of 0.25 (Lee et al., 2011; Risso et al., 2011). Normalized samples were batch-corrected in order to remove artifacts from well-plates and/or the sequencing center. The batch correction was performed using *Combat* (Johnson et al., 2007) (40), implemented in the R-package, *SVA* (Leek et al., 2012). In-house R scripts were subsequently used to extract the processed data from *MZF1* transcripts and for the transcripts of its interactors. We carried out the analyses using all samples within a tumor-type, as well as matched paired samples only (i.e., tumor and normal samples from the same patient), to evaluate how this would potentially affect results. The normalized counts were log2 transformed (pseudo count = 1) to overcome the problem of extreme values due to differences in sequencing depth (Lee et al., 2011).

We then incorporated the gene expression data in the MZF1 protein-protein interaction network using *Cytoscape* version 3.3.0 (Shannon et al., 2003). In each network, the absolute value of the difference between the medians of the counts per gene in the normal samples and tumor samples was used to represent the node colors, upon log2 transformation. The color shade of the edges represented the value of the Pearson correlation coefficient calculated for each MZF1 interactor-pair according to the counts presented in the tumor samples with respect to the normal samples.

### Identification of cancer mutations and prediction of cancer mutation effects

We collected a subset of cancer-related mutations known to be located in the MZF1 SCAN domain [residues 35–128, PDB entry 2FI2 (23)] from different cancer genomics databases including *Cbioportal* (Cerami et al., 2012), *COSMIC* (Forbes et al., 2015), *CMPD* (Huang et al., 2015), *ICGC* Portal (Hudson et al., 2010), and *CancerResource* (Ahmed et al., 2011) databases. We also inferred additional MZF1 mutations from *HumanSavar* (http://www.uniprot.org/docs/humsavar), *CanProVar* (Li et al., 2010), *1000 Genomes* (Auton et al., 2015), and *dbSNP* (Sherry et al., 2001), which all report mutations that have not been associated with disease and their clinical relevance is still unknown.

### Sequence-based prediction of functional impact of MZF1 mutations

Prediction of the functional effects of mutations was carried out using different sequence-based methods. Specifically, we integrated *Provean* (Choi et al., 2012), *Mutation Assessor* (Reva et al., 2011), *Polyphen2* (Adzhubei et al., 2013), *PON-P2* (Niroula et al., 2015), *SNAP2* (Bromberg et al., 2008), *AlignGVGD* (Tavtigian, 2005; Mathe et al., 2006), and *MutPred* (Li et al., 2009). Each method had different threshold values to discriminate between pathogenic and neutral mutations. Thus, to compare them we applied the cutoffs associated with each of them in the original publications. Specifically, we used threshold values < −2.5 (*Provean*), >2 (*Mutation Assessor*), >0.5 (*Polyphen2* and *PON-P2*), <0 (*SNAP2*), and >0.75 (*MutPred*) to discriminate between the deleterious/partially deleterious and the neutral mutations. We considered a mutation as “damaging” if it was classified as being within C45, C55, and C65 classes of *AlignGVGD*.

### Structure-based prediction of impact on protein stability and binding interface

We employed the *FoldX* energy function from the newest release of the *FoldX suite* (27) to carry out *in silico* saturation mutagenesis using a *Python* wrapper that we recently developed (*to be published and available on request*). The wrapper allows for the introduction of all possible 19 point mutations at each position of the protein using multithread calculations. Calculations with the wrapper resulted in an average ΔΔG (differences in ΔG between mutant and wild type variant) for each mutation over the whole NMR ensemble of 20 conformers for both the MZF1 monomer (PDB entry 2FI2, chain A or B only) and the dimer (PDB entry 2FI2, both chain A and B). The ensemble was used to account for flexibility in the protein since *FoldX* energy function only allows for local conformational changes. We calculated the ΔΔG between mutants and wild type variants associated with protein stability and in relation to the formation of the dimeric MZF1 complex. We applied the *BuildModel* module from the *FoldX suite* and five independent runs for mutations in our scan. The typical prediction error of FoldX is about 0.8 kcal/mol (Guerois et al., 2002). Twice the prediction error (i.e., 1.6 kcal/mol) was used as a threshold to discriminate between neutral and deleterious mutations in the analyses.

Moreover, we implemented a correction to the *FoldX* energy values, as defined by Tawfik's group (Tokuriki et al., 2007) to make the ΔΔG *FoldX* values more comparable with the expected experimental values (i.e., to achieve more overlapping distributions). We estimated the ΔG of unfolding for each mutant variant (ΔGu_mutant_) from the equation:

(1) $$\begin{array}{r} \Delta {\text{Gu}}_{mutant}=\Delta {\text{Gu}}_{WT\text{\_}exp}\;+\;m^{*}\Delta \Delta {\text{G}}_{Foldx}+\;b \end{array}$$

where ΔGu_*mutant*_ is the estimated unfolding ΔG of the mutant variant, ΔGu_*WT*_*exp*_ is the experimental unfolding ΔG of the wild type variant, ΔΔG_*Foldx*_ is the ΔΔG estimated by *FoldX, m* and *b* are correction terms. In particular, *m* and *b* where selected according to the work by Tawfik et al. (Tokuriki et al., 2007), in which the correlation between the experimental values ΔΔG values and those predicted by *FoldX* was calculated for 1285 mutations of 10 different proteins available in the *ProTherm* database (Kumar, 2006). The authors applied a linear correction to the *FoldX* ΔΔG values to optimize the overlap between the distributions of the experimental and *in silico* data, using parameters (*m* and *b*) derived either from linear regression or Principal Component Analysis (PCA). The latter correction led to essentially identical distribution between the experimental and computational data, therefore all the *FoldX* values were corrected using the PCA equation ΔΔG_*FoldX*_ = −0.078 + 1.14 ΔΔG_*Experimental*_ (Tokuriki et al., 2007). Therefore, we here used the inverse of this relation, from which *b* and *m* were derived, to normalize our *FoldX* ΔΔGs before adding them to the experimental unfolding free energies. Reversing the equation ΔΔG_*FoldX*_ = − 0.078 + 1.14 ΔΔG_*Experimental*_, we obtained:

(2) $$\begin{array}{r} \Delta \Delta {\text{G}}_{Experimental}\;=\;0.078+0.877\;\Delta \Delta {\text{G}}_{FoldX} \end{array}$$

Where *b* = 0.068 and *m* = 0.877 to be used in Equation (1).

In our calculations, we cannot use an experimental value of unfolding ΔG for the wild-type variant of MZF1 SCAN domain since there are no experimental data available in the literature at the best of our knowledge. Nevertheless, values in the range of 5–15 kcal/mol are generally obtained for the net free energy of unfolding of proteins (Privalov, 1979; Fersht and Serrano, 1993). And other helical proteins have folding ΔG ~5–7 kcal/mol according to the data reported in *ProTherm*. We thus here used as an arbitrary reference a value of 5 kcal/mol as ΔGu_*WT*_*exp*_ in the calculation of ΔGu_*mutant*_.

### Generation of the MD ensembles

We used the first conformer of the NMR ensemble (PDB entry 2FI2, Peterson et al., 2006) as a starting structure for all-atom explicit solvent MD simulations with Gromacs 4.6 (Hess et al., 2008). We carried out MD simulations with five different force fields belonging to different force field families to assess the robustness of the results, i.e., Amber-ff99SB^*^-ILDN (Best and Hummer, 2009; Lindorff-Larsen et al., 2010), Amber-ff99SB-NMR-ILDN (Li and Brüschweiler, 2010), CHARMM22 with the CMAP backbone corrections (herein termed CHARMM27; Mackerell et al., 2004; Bjelkmar et al., 2010), CHARMM22^*^ (Piana et al., 2011), and the modified OPLS-AA/L force field RSFF1 (Jiang et al., 2014). We used TIP3P adjusted for CHARMM force fields (MacKerell et al., 1998) and TIP4P-Ew (Horn et al., 2004) water model for Amber/CHARMM and RSFF1 force fields, respectively. The protein was solvated in a dodecahedral box with a minimum distance between protein and box edges of 1.2 nm applying periodic boundary conditions. His51 was simulated as the Nε2-H tautomer, according to NMR CD2-HD2 chemical shift (BMRB accession number: 6957) which is <122 ppm (Sudmeier et al., 2003). The system was equilibrated according to a protocol previously applied to other cases of study (Papaleo et al., 2014b; Tiberti et al., 2015a). Productive MD simulations were carried out in the canonical ensemble at 298 K using velocity rescaling with a stochastic term (Bussi et al., 2007). The LINCS algorithm (Hess et al., 1993) was used to constrain the heavy atom bonds to use a time-step of two fs. Long-range electrostatic interactions were calculated using the Particle-mesh Ewald (PME) summation scheme (Essmann et al., 1995). Van der Waals and Coulomb interactions were truncated at 0.9 nm.

### Comparison of MD ensembles with NMR data

We selected 501 frames, which were equally spaced in time, from each of the five different MD simulations to calculate backbone and side-chain chemical shifts, as well as NOEs. We used backbone and side-chain chemical shifts for 90 residues. Moreover, we employed 4063 NOE-derived distances of which 1018 long-range (i.e., separated by more than four residues in the primary sequence), 2422 short-range, and 623 intermolecular.

For each of the MD frames, we predicted the chemical shift using *PPM One* (Li and Brüschweiler, 2015), after which we compared the predicted chemical shift with the experimentally-derived values (deposited in BMRB entry 6957) using a χ^2^–like approach according to the following equation:

$$\begin{array}{l} \chi^{2}\;=\;\frac{1}{s-1}\sum_{j=1}^{s}\left(\frac{{\left(\frac{1}{n}\sum_{i=1}^{n}\delta_{i,j}-\delta_{exp}\right)}^{2}}{\delta_{exp}}\right) \end{array}$$

We calculated the pair-wise distances between the atoms for which experimental NOEs were available and then averaged them over the 501 frames extracted from each MD ensemble. Differences between average and experimentally obtained distances were then calculated.

For each MD ensemble we also predicted the resolution value using *Resprox* (Berjanskii et al., 2012) and compared it to the predicted resolution of the NMR ensemble deposited in the PDB (PDB entry 2FI2).

### Network analyses of MD ensembles

A protein structure network (PSN) approach was used as implemented in the *Pyinteraph* framework (Tiberti et al., 2014). The residues that have zero edges are termed as “orphans” and those involved in more than three edges are referred as “hubs.” The node inter-connectivity was used to identify the so-called “connected components,” i.e., a cluster of connected residues in the graph. The node clustering procedure was carried out so that each node was iteratively assigned to a cluster if the node could establish at least a link with another node of the same cluster. We tested two different distance cutoffs to define the existence of a link between the nodes (i.e., 0.5 and 0.55 nm). We then monitored the distribution of the hubs and the elements belonging to the first five more populated connected components to identify a suitable cutoff for the analyses. The distance is measured between the centers of mass of the residue side chains, except for glycine residues (these are not included in the analysis). Since MD force fields are known to have a different mass definition, we used *PyInteraph* mass databases for each of the MD ensembles.

To obtain a single PSN for each MD ensemble, we included, in the final graph, only those edges which were present in at least 20% of the simulation frames (*p*_*crit*_ = 20%), as previously applied in other cases of study (Papaleo et al., 2012a,b, 2014a; Jónsdóttir et al., 2014; Tiberti et al., 2014; Lambrughi et al., 2016a,b). For each pair of nodes in the PSN graph, a variant of the depth-first search algorithm was employed to identify the shortest path of communication. The distance between two nodes (i.e., residues) that are directly connected in the graph was considered to be one. The shortest path was defined as the path in which the two residues were non-covalently connected by the smallest number of intermediate nodes. The calculations were performed using the *PyInteraph* suite of tools (Tiberti et al., 2014) and analysis of the output was performed using in-house *Python* scripts (available on request). To evaluate the convergence of the PSN properties over the simulation time, we used the Jack-Knife resampling method (Miller, 1974). In particular, we calculated the hubs and connected components from an average PSN generated by the whole MD ensemble and by smaller MD ensembles obtained discarding 10% of the simulation frames at regular time intervals.

## Results and discussion

### The landscape of protein-protein interactions mediated by MZF1 SCAN domain

**Figure 1:**
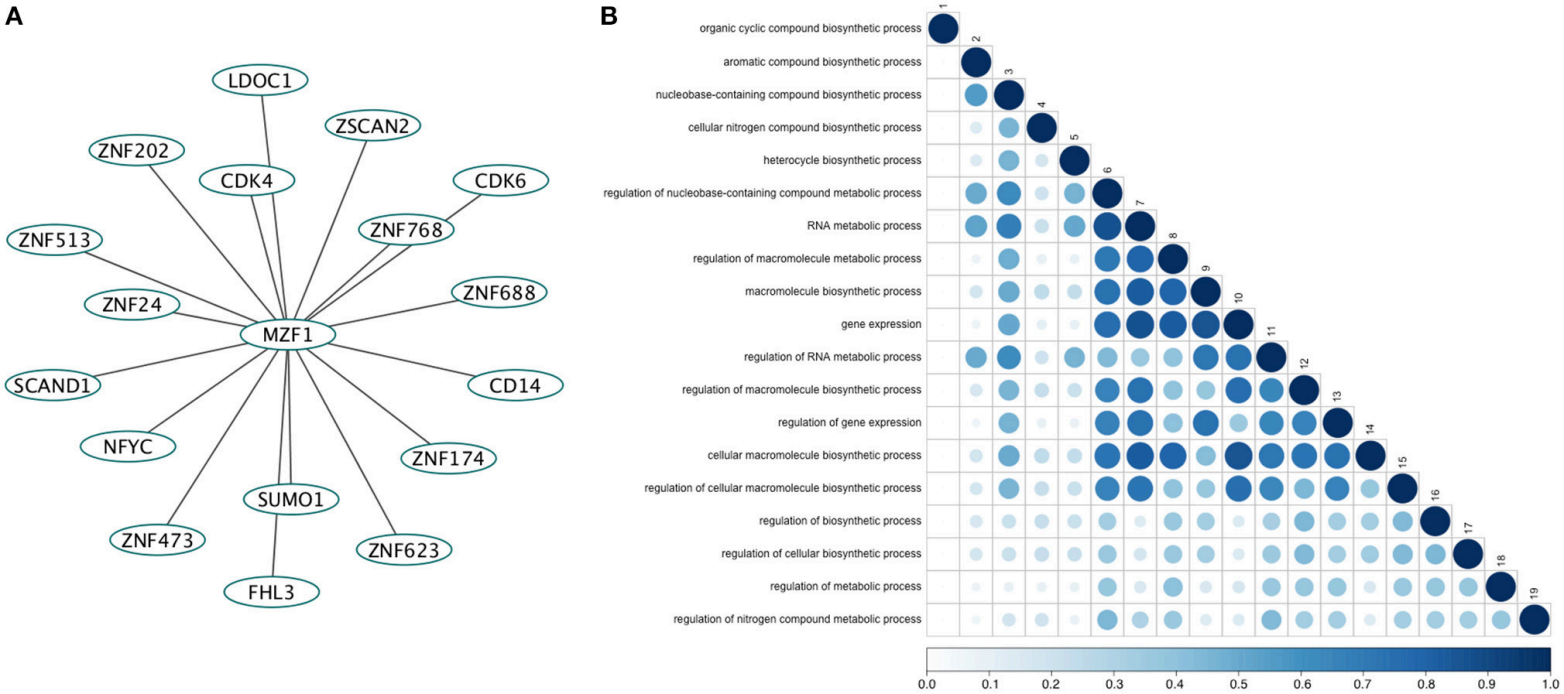
**(A)** The direct network of MZF1 protein-protein interactions. Interaction partners of MZF1 were extracted from the *Interologous Interaction Database* (*I2D*). **(B)** Results of gene ontology (GO) enrichment analysis. Similarity of GO-terms was estimated using the R-package *GOSemSim* (Yu et al., 2010). The shade and size of dot indicates relevance (strength) of similarity. The diagonal is similarity with self. Similarity scores range from 0 to 1.0.

We retrieved 17 MZF1 interaction partners from the *I2D* database (Figure 1A). For each of them we collected information on the experimental techniques used to probe the interaction and the presence of SCAN or zinc finger domains (Table S2). Among the identified interactors, five proteins (ZNF202, ZNF24, ZNF174, and ZSCAN2) were SCAN-containing and harbored multiple zinc finger motifs, while only SCAND1 was a SCAND protein. Gene ontology analyses of the MZF1 interaction network pointed to a major role in gene transcription as well as in the regulation of other biosynthetic processes (Figure 1B).

**Figure 2:**
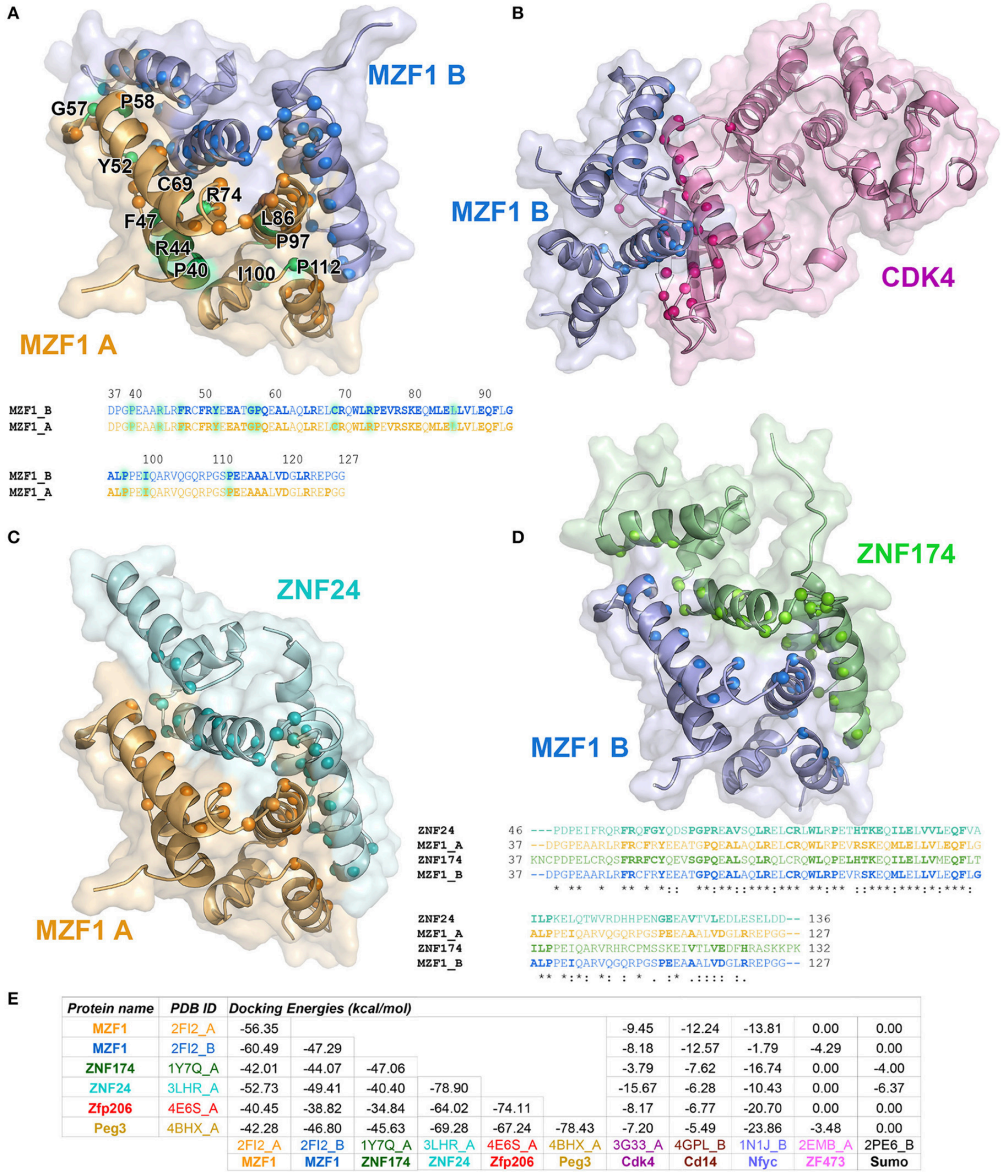
**Predicted protein-protein interactions mediated by MZF1 SCAN domains**. The predicted homo- and hetero-dimeric complexes of MZF1 SCAN domain (PDB entry 2FI2 chain A orange, chain B blue) and ZNF174 SCAN domain (PDB entry 1Y7Q chain A green) ZNF24 SCAN domain (PDB entry 3LHR chain A cyan) and Cdk4 (PDB entry 3G33 chain A violet) are represented as cartoon and surface in **(A–D)**, respectively. We calculated the residues of the two proteins in each complex that have at least one atom within 0.4 nm of distance from the binding partner and we highlighted their Ca as spheres. These residues are shown in bold in the amino acid sequences of the SCAN domains. **(A)** Predicted homo-dimer of MZF1 SCAN domain with indicated in green the position of the deleterious mutations that we identified. The amino acid sequence of MZF1 SCAN domain is reported. **(B)** Predicted hetero-dimer of MZF1 SCAN domain and Cdk4 (blue and violet, respectively). **(C)** Predicted hetero-dimer of MZF1 and ZNF24 SCAN domains (orange and cyan, respectively) **(D)** Predicted hetero-dimer of MZF1 and ZNF174 SCAN domains (blue and green, respectively). The amino acid sequence of the MZF1/ZNF24 and MZF1/ZNF174 SCAN domain complexes are reported and the residues at the interaction interfaces are shown in bold. **(E)** *PRISM* docking energies for the dimeric complexes between MZF1 and MZF1 partners from the *I2D* database and with Peg3 and Zfp206 SCAN domains. PRISM predicts with similar energies three different SCAN template interfaces (2fi2AB, 3lhrAB, and 1y7qAB) for nearly all the predicted homo- and hetero-complexes involving SCAN domains, as for example in the case of the homodimer of Znf174 where the three interfaces have docking energy values ranging from −47 to −45 kcal/mol. In the table we reported the predicted interaction complexes with lowest energies for each pair of interactors.

To identify those interactors, which form a complex with the MZF1 SCAN domain, we employed the *PRISM* approach. As a target ensemble for *PRISM*, we used the structures of each of the MZF1 SCAN domain monomers (PDB entry 2FI2, chain A and chain B). Moreover, we used the interaction partners, from *I2D*, for which at least one experimental structure was available in the *PDB*. We also included two additional SCAN domain proteins, the Zfp206 and Peg3 [PDB entries 4E6S (Liang et al., 2012) chain A and 4BHX (Rimsa et al., 2013) chain A, respectively] to further investigate the role of SCAN-mediated hetero-dimerization. For analyses, we retained only those complexes with a predicted docking energy lower than −2.39 kcal/mol (Figure 2).

The *PRISM* analyses suggest that all the SCAN domains, and MZF1 especially, can form homo- and hetero-dimers with other SCAN domains with a very low predicted docking energy (< −33.46 kcal/mol; Figure 2E). These results are confirmed by the fact that the docking energy for MZF1 homo-dimerization, for which the structure is known (PDB entry 2FI2), is within the same range of other homo- and hetero-dimeric complexes of SCAN domains predicted by *PRISM*. All the predicted homo- and hetero-complexes of SCAN domains have similar interaction interfaces mainly comprising residues in the α-helices 2, 3, and 5 (residues 58–73, 80–95, 112–123 from PDB entry 2FI2). Residues known to contribute to MZF1 homo-dimerization are also conserved at the interface of other SCAN-SCAN complexes predicted by *PRISM*, such as F47, R48, Y52, P58, A61, L65, R66, W72, L73, P75, K82, L84, V88, Q93, P97 (2FI2 numbering, Figure 2). Interactions with the Cdk4 kinase, CD14, and the Nfyc transcription factor are also predicted within the significance threshold, suggesting an even broader network of interactions and diversity in signaling and expression regulation, promoted by the SCAN module.

### Expression levels of MZF1 and its interactors in 24 cancer studies from the Cancer Genome Atlas (TCGA)

To evaluate the relationship between the MZF1 network (i.e., MZF1 and its interactors) and different types of cancer, we analyzed genomic profiling data from 10173 samples stratifying into 24 different cancer studies—deposited in *TCGA* (Table S1).

**Figure 3:**
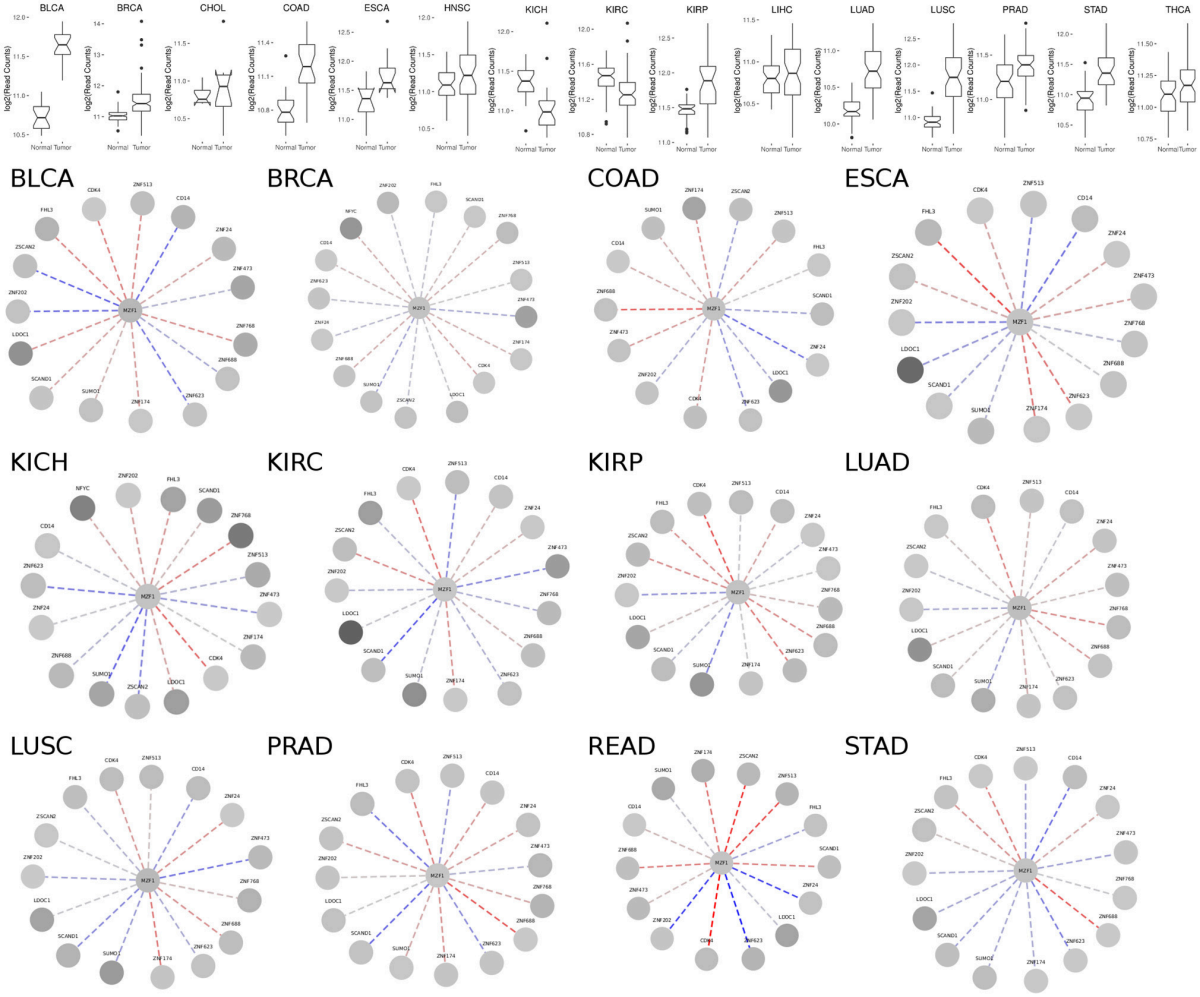
**(Upper panel)** Comparison of *MZF1* expression levels as determined by RNASeq experiments comparing paired tumor and normal samples of patients from different cancer studies deposited in *TCGA*. **(Lower panel)** Correlation between changes in the expression levels of *MZF1* and its interactors as determined by RNASeq experiments comparing paired tumor and normal samples. In each network, the absolute value of the difference between the medians of the counts per gene in the normal samples and tumor samples was used to represent the node colors, upon log2 transformation. The color shade of the edges represented the value of the Pearson correlation coefficient calculated for each MZF1 interactor-pair according to the counts presented in the tumor samples with respect to the normal samples. Red and blue dotted lines show positively and negatively correlated pairs, respectively.

*MZF1* is expressed at an overall higher or lower level in tumor vs. normal samples in 19 out of the 24 cancer studies that we analyzed, strongly supporting its crucial but diverse role in cancer (Figure 3, Figure S1 and Table S1). The majority of cases are characterized by higher levels of *MZF1* within tumors, with the exception of Adrenal Gland and KICH/KIRC Kidney tumors, in which *MZF1* has lower expression levels in tumor compared to normal samples. In many cancer types we observed a high variability in the expression levels of *MZF1*, emphasizing the importance of using paired tumor-normal samples in the comparisons. We then investigated if any correlation was observed between *MZF1* changes and changes in the expression levels of its interactors. We observed as a common feature that changes in *CDK4* and *ZNF688* positively correlate (red dotted lines in the Figure 3) with changes in *MZF1* (Figure 3). MZF1 has a predicted phosphorylation site for CDK kinases in its N-terminal region (Ser8) and we thus hypothesize that the MZF1 SCAN domain acts as a docking site for CDK4 kinase, which is in turn able to recruit the MZF1 disordered N-terminal tail for phosphorylation. The CDK4 binding site on MZF1 predicted by *PRISM* (Figure 2) partially overlaps with the SCAN-SCAN dimerization interface and thus the binding to the kinase would compete with the dimer formation according to our models.

We also noticed that the correlations between expression levels of *MZF1* and the other SCAN domains vary from one cancer type to the other, unveiling a complex network of interactions and diverse cellular signaling that can be elicited in different cancers. The SCAND-only SCAND1 protein has been experimentally reported to interact with MZF1 and its expression levels and *MZF1* expression levels are tightly correlated in the majority of the cancer types according to our analyses (Figure 3). SCAND1 is thus likely to act as a regulator of MZF1 activity, in agreement with the hypothesis that SCAND1 could decrease MZF1-mediated signaling by altering its affinity for DNA targets (12). In certain cancer types, when *MZF1* level increases, *SCAND1* is also up-regulated, whereas in other cancer types they are inversely correlated, i.e., *MZF1* levels increase but *SCAND1* expression decreases compared to the normal samples. The latter scenario occurs in KIRC, LUSC, PRAD, and STAD cancer studies annotated in *TCGA* (Table S1, Figure 3). In these cases, the regulatory function of SCAND1 on MZF1 activity is likely to be lost altering the downstream effects mediated by this transcription factor. The same heterogeneity in different cancer types is observed for the other SCAN-containing proteins.

### Sequence-based prediction of the effects induced by mutations in the MZF1 SCAN domain

Twenty three cancer-related mutations located within the MZF1 SCAN domain were identified through the analyses of various cancer databases (see Section Materials and Methods) along with 21 mutations which have not been associated with cancer so far (Table S3).

We subsequently employed both sequence- and structure-based methods to predict the impact of these mutations on function, stability and protein-protein interactions.

**Figure 4:**
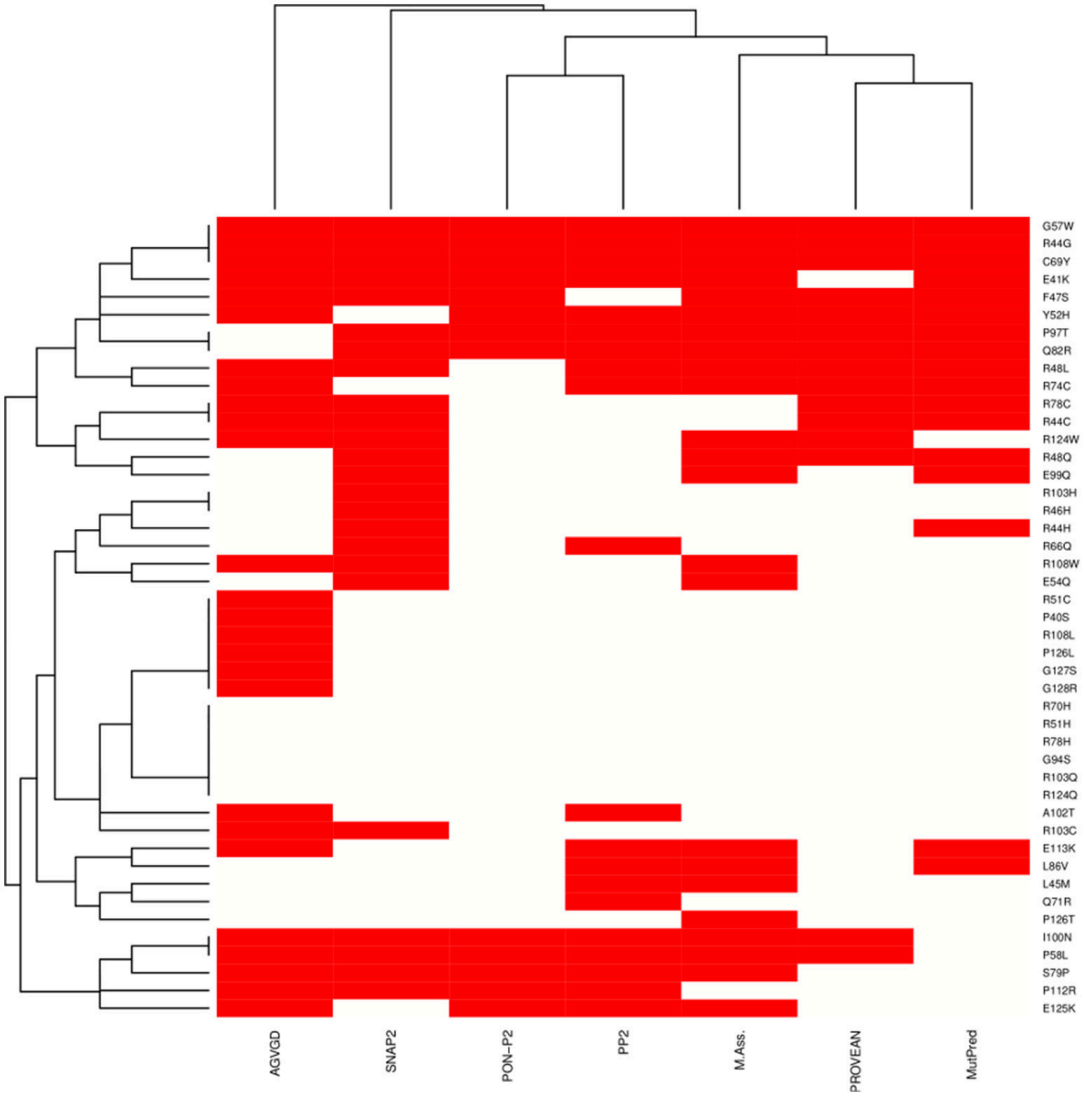
**Heat-map and clustering of the effects induced by MZF1 mutations on protein function as predicted by sequence-based classifiers**. Seven sequence-based methods have been employed for the prediction, i.e., *Align-GVGD* (AGVGD), *SNAP2, PON-P2, Polyphen2* (PP2), *Mutation Assessor, PROVEAN, MutPred*. Deleterious and neutral mutations are depicted in red and white, respectively. A complete consensus is observed only for a small fraction of the mutations. Nevertheless, the different methods are in reasonable agreement, with most of the mutations showing consensus for five out of seven methods.

We integrated seven sequence-based methods mainly based on evolutionary information to assess the pathogenic potential of the mutations in the MZF1 SCAN domain. The methods employed were in agreement for most of the mutations even if a complete consensus could only be identified for nine mutations (Figure 4). *MutPred, Provean PON-P2, Polyphen 2*, and *Mutation Assessor* predictors yielded similar results, as illustrated by the hierarchical clustering reported in Figure 4. A subset of predicted deleterious amino acidic substitutions included E41K, R44G, F47S, R48L, Y52H, G57W, P58L, C69Y, R74C, S79P, Q82R, P97T, and I100N. In addition, most methods (five out of seven) identified another group of substitutions with neutral effects on protein function i.e., P40S, R51C, R51H, R70H, R78H, G94S, A102T, R103C, R103Q, R108L, R124Q, P126L, P126T, G127S, and G128R. *MutPred* also predicted with significant *p*-values (*p* < 0.05) a gain or a loss of Post-Translational Modifications (PTMs) upon certain mutations, such as gain of glycosylation in E41K (assuming that the lysine is hydroxylated), gain of phosphorylation at F47S and loss of methylation at R48 and R51.

### Effects of MZF1 mutations on structural stability and dimer formation of the SCAN domain

**Figure 5:**
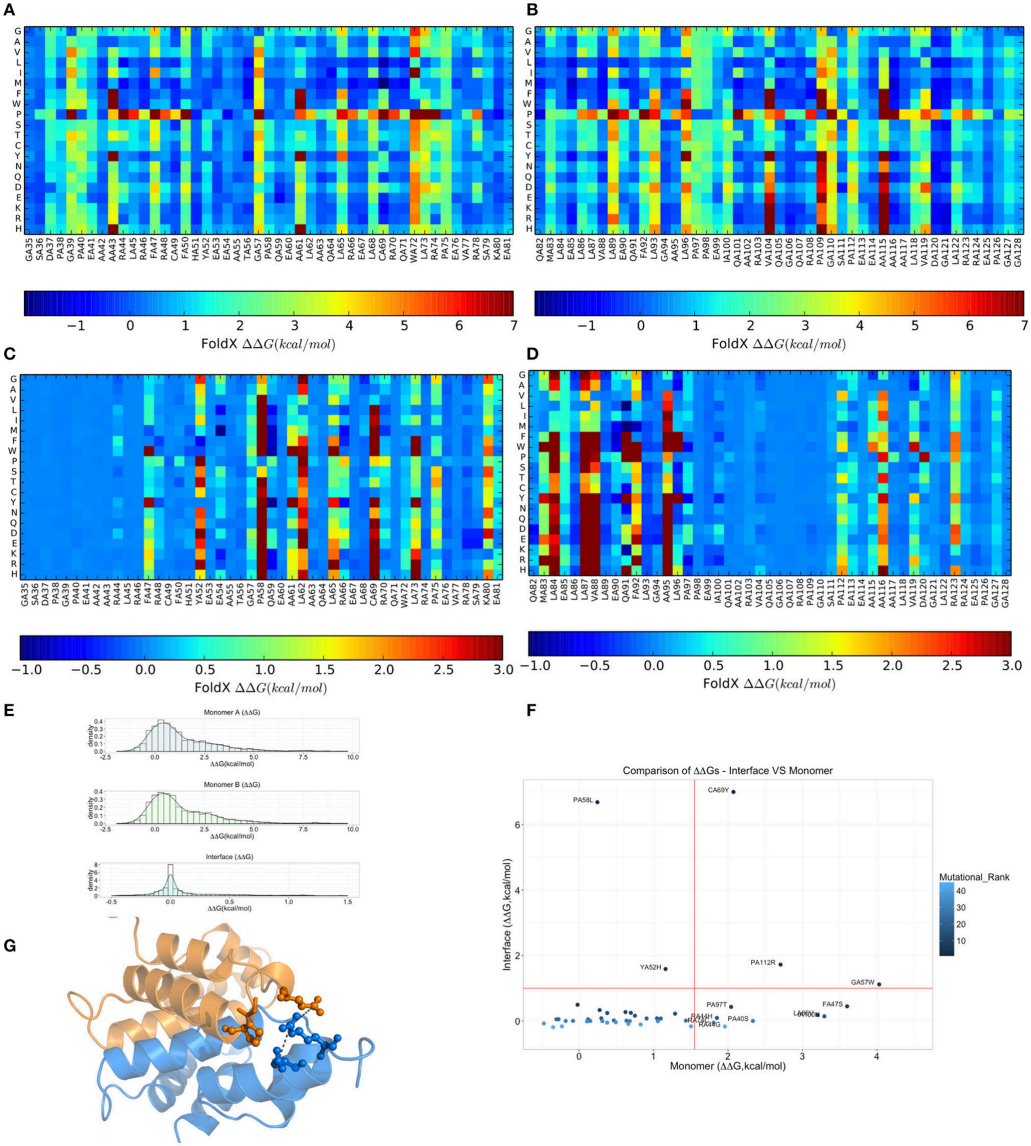
**Effects on thermodynamic protein stability and protein-protein complexes upon ***in silico*** saturation mutagenesis of MZF1 SCAN domain. (A–D)** Heatmap based on calculations of ΔΔG associated with monomer protein stability **(A,B)** and monomer-monomer binding **(C,D). (E)** The distribution of the ΔΔG values from saturation mutagenesis of the monomer or dimer MZF1 SCAN domain is shown. **(F)** ΔΔG predictions for mutations in MZF1 SCAN domain that have been deposited in cancer databases and other databases of genetic variations. **(G)** Structural constraints induced by P58 rigid side chain promote a cluster of electrostatic interactions at the dimerization interface.

Due to the intrinsic limitations in sequence-based methods to predict mutational impact, we turned our attention to structure-based methods. Specifically, we carried out an *in silico* saturation mutagenesis scan based on the *FoldX* energy function using the whole NMR conformational ensemble of the MZF1 SCAN deposited in the PDB (Figure 5). The *FoldX* energy function provides a quantitative description of the intermolecular interactions that stabilize a protein in order to predict the change in thermodynamic folding stability or in the free energy of protein complex formation (ΔΔG) with respect to the wild type. The saturation scan allowed us to assess the impact of the mutations on protein structural stability (the scan is performed on the MZF1 monomers) and local effects influencing the interface for monomer-monomer interaction (when the scan is performed on the MZF1 dimer). Negative ΔΔG values indicate variants that are more stable than the wild type, whereas positive values indicate that the mutant variants are less stable than the wild type MZF1 protein. Thus, mutant variants with ΔΔG >0 upon the monomer scan have a higher population of (partially) unfolded structures that are prone to aggregation, misfolding or degradation. Mutant variants with ΔΔG >0 upon the protein-protein binding scan have a decreased monomer-monomer binding affinity. The high-throughput mutation scan allowed us not only to assess the impact of MZF1 mutations collected from the mutation databases, but also to predict the effect of any other possible amino-acid substitution (Figures 5A–D). Thus, the full data set provides a valuable source of information such as (i) the predicted impact of MZF1 mutations potentially identified in future studies related to disease and (ii) critical hotspots for protein structural stability and/or protein-protein binding. The data sets comprised 1786 and 3572 mutated variants for monomer and dimer scans, respectively. The distribution of ΔΔG values for the data sets (Figure 5E) is similar to those of other proteins with different folds investigated using the same pipeline (Papaleo et al., 2014a; Mathiassen et al., 2015). The distribution of ΔΔG values for complex formation turned out to be much narrower with values rarely higher than 5 kcal/mol. This observation may be partially explained by the fact that the *FoldX* energy function is only capable of accounting for local effects of mutations. Indeed, as there are no major conformational changes of the backbone during *FoldX* calculations, the method will neglect the contribution of mutations located at distal sites with respect to the monomer-monomer interface. The heatmaps depicting the results of the saturation scan are reported in Figures 5A–D, while the analyses of the MZF1 mutations reported in online databases (Table S3) are shown in Figure 5F. Based on Figure 5F, the only substitutions which did not affect protein stability but had a marked impact on the dimer formation were P58L and Y52H. These two sites are predicted to be sensitive to any amino acid substitution at the interface, as observed from the heatmap (Figures 5C,D). A proline at position 58, due to the intrinsic rigidity of its side chain, is crucial for the proper orientation of the upstream turn motif. This motif allows a glutamate (E54) within one of the monomers (blue in Figure 5G) to participate in an electrostatic intermolecular network with the residues R123 and D120 of the other monomer (orange in Figure 5G). Our scan also pointed to a subset of mutations with detrimental effects on both protein stability and the formation of the dimeric complex e.g., C69Y, P112R, and G57W. The majority of the remaining deleterious mutations including F47S, I100, L86V, P97S, P40S, R44G, R44H, and R74C mainly had an impact on protein stability. The deleterious amino acidic substitutions mentioned above were also located in sensitive structural hotspots where most substitutions are not tolerated, as seen by the heatmap (Figures 5A,B).

To better appreciate the effects of these mutations on protein stability, we also estimated a ΔG of unfolding (Δ*Gu*_*mutant*_) of the mutant variants reported in Table S3, as explained in details in the Materials and Methods. The Δ*Gu*_*mutant*_ values are also reported in Table S3. These data could be useful for comparison with future experimental determination of changes in the free energies of folding/unfolding upon mutation of MZF1 and overall confirm the scenario described above.

Moreover, we noticed that the mutations predicted to be damaging for protein structure and function had previously been identified in cancer studies, as well as in databases that contain mutations not yet classified for their clinical relevance (Table S3). Our findings suggest that these unclassified mutations could exert damaging effects on MZF1, highlighting the need for further experimental studies and greater efforts in genomic profiling of cancer patients. Collectively, our results demonstrate the utility of structure-based computational tools to discriminate between substitutions more likely to be cancer drivers and those exerting only neutral effects. F47S, G57W, and C69Y are of particular interest as these were predicted to affect protein stability or dimerization interface and have previously been identified in patients with bladder cancer, kidney cancer, colorectal cancer, and in established cancer cell lines (Table S3).

### Validation of MZF1 MD ensembles with NMR data

One of the limitations of the high-throughput saturation mutagenesis performed above is that only local structural changes can be modeled. Thus, the information on structural changes promoted by distal sites are lacking. To overcome this issue, we carried out microsecond all-atom explicit solvent MD simulations using state-of-the-art force fields. We accounted for differences in the formulation of the physical models employed in MD on the simulated dynamics, using five different force fields, i.e., CHARMM22^*^, CHARMM27, Amber99-SB^*^-ILDN, Amber99-SB-NMR-ILDN, and RSFF1. Even minor changes in the torsional potential of protein backbone and side chains in the force fields have a major impact on the dynamics and structure described by MD simulations (Guvench and MacKerell, 2008; Lange et al., 2010; Lindorff-Larsen et al., 2012; Martín-García et al., 2015; Tiberti et al., 2015b; Unan et al., 2015) and the structural consequences upon selecting a certain set of force field parameters are hard to predict.

The MZF1 SCAN domain has been studied by NMR spectroscopy resulting in a number of high quality probes of protein dynamics in solution, such as backbone and side-chain chemical shifts, as well as short- and long-range NOEs (Peterson et al., 2006). These data are a valuable resource for experimental validation of the conformational ensembles collected in our MD simulations as well as for comparison of MD force fields (Guvench and MacKerell, 2008; Lange et al., 2010; Beauchamp et al., 2012; Best et al., 2012; Dror et al., 2012; Lindorff-Larsen et al., 2012; Piana et al., 2014; Papaleo et al., 2014b; Henriques et al., 2015; Martín-García et al., 2015; Papaleo, 2015; Unan et al., 2015; Tiberti et al., 2015b). Thus, we calculated these NMR parameters from each MD ensemble and compared them to the experimental values.

**Figure 6:**
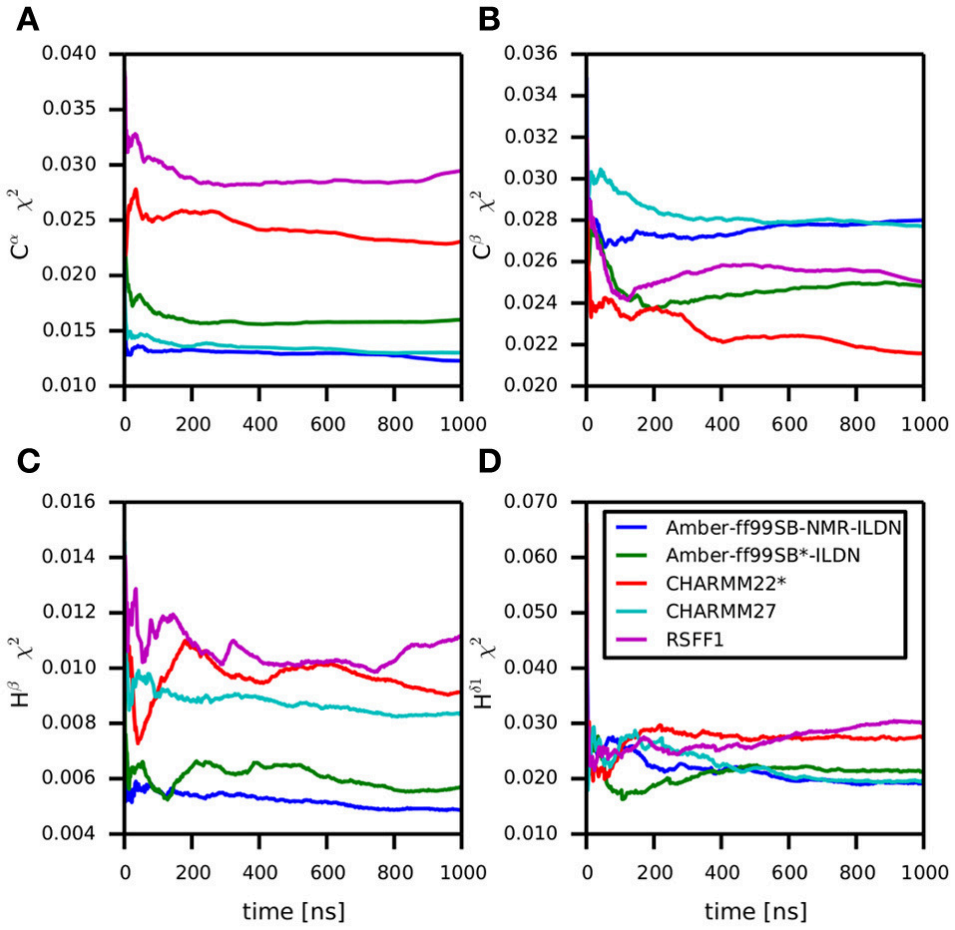
**Differences between predicted chemical shifts from MD simulations and experimentally measured chemical shifts along the simulation time**. All the simulations are largely in agreement with experimentally derived chemical shifts. Indeed, the MD ensemble converge to very low deviation from the experiments after 200–300 ns of simulation. The chemical shift for different backbone atom types are shown in the panels **(A–D)**.

**Figure 7:**
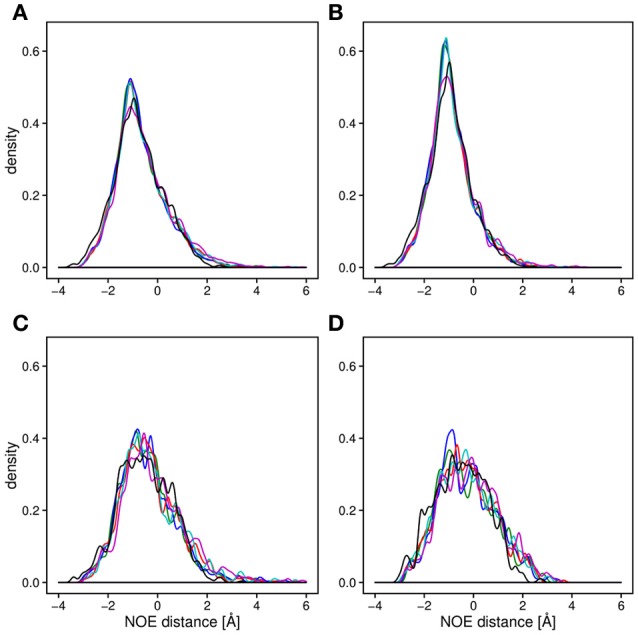
**Density plot of the average distance difference of NOE pairs between MD simulations and measured NOEs**. For all the panels, the distances were averaged over the trajectory and subtracted from the measured NOE (see Section Materials and Methods). The distance differences between the all measured NOEs **(A)** are very similar for all of the force fields (black; NMR conformers, blue; Amber-ff99SB-NMR-ILDN, green; Amber-ff99SB^*^-ILDN, red; CHARMM22^*^, cyan; CHARMM27, magenta; RSFF1) as well as for the 20 NMR conformers (PDB:2fi2). Noticeable is a slightly lower average distance between the NOE pairs compared to the experimentally obtained values. The similarity between the force fields is observed when plotting only long range NOEs **(B)**, short range NOEs, **(C)** as well as the intermolecular NOEs **(D)**.

Backbone and side-chain chemical shifts are known to report on motions occurring on a heterogeneous range of time scales (Robustelli et al., 2012; Case, 2013; Palmer, 2015). The calculated chemical shifts from our simulations are in good agreement with the experimental values as it can be appreciated by the low χ^2^ values (Figure 6, Figures S2, S3). They converge rather quickly during the simulation time (after ~100–200 ns). NOEs can be used to report on either secondary structure (short-range NOEs) or tertiary contacts (long-range NOEs). Also in this case we did not observe remarkable deviation with respect to the original NMR ensemble, suggesting that the selected MD force fields describe with good accuracy the structure and dynamics of MZF1 (Figure 7).

The similarity between the different MD ensembles was also highlighted by the overlap between the first 20 principal components of the covariance matrix of Cα atomic fluctuations estimated in terms of Root Mean Square Inner Product (RMSIP; Hess, 2002). RMSIP was continuously higher than 0.85 for any pair-wise comparison of the MZF1 MD ensembles, suggesting a high overlap between the conformational spaces described by our simulations.

**Figure 8:**
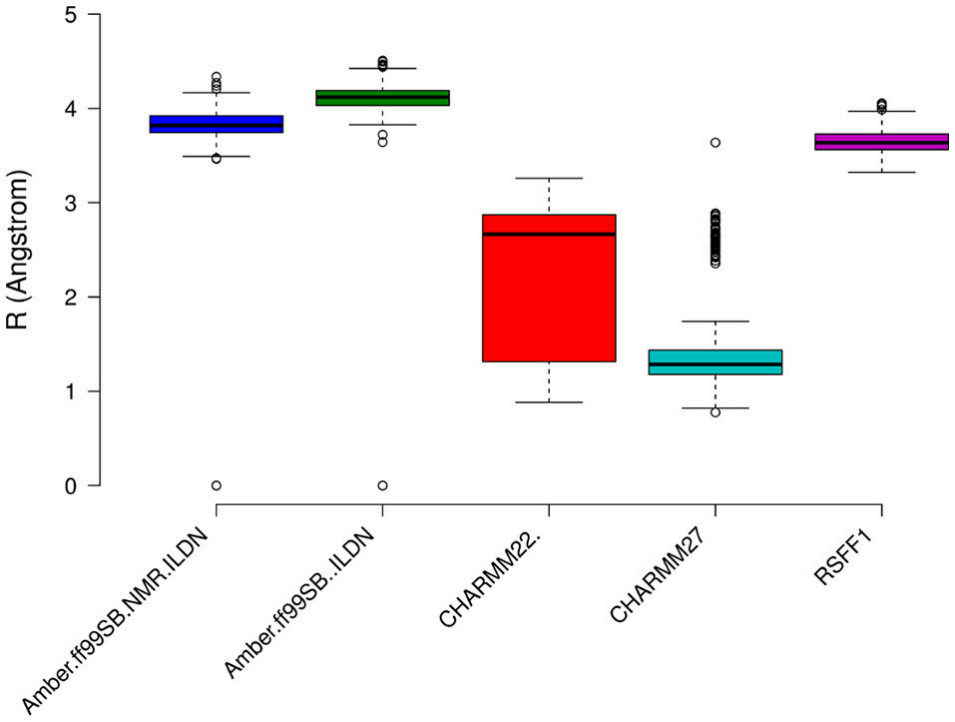
**Prediction of Resolution values for the different MD ensemble of MZF1 dimer generated using different atomistic force fields**. A χ^2^-like score was used to estimate the differences between experimental and computationally derived chemical shifts as detailed in the Section Materials and Methods.

The prediction of structural resolution (R) for the MD ensemble (Figure 8) allowed us to better discriminate between the different force fields. We observed a shift to lower R values and thus higher structural quality in MD ensemble with the CHARMM force-field family. The predicted *R*-value quantitatively accounts for different structural quality parameters (Berjanskii et al., 2012) such as population of χ_1_ side-chain dihedral angles, side-chain rotamers that lie outside the distribution described by the penultimate rotamer library (Lovell et al., 2000), outliers in the Ramachandran plot, packing of the protein core, hydrogen-bond networks and atomic clashes. Thus, the predicted *R*-value is sensitive toward different structural properties with respect to chemical shifts and NOEs and is a powerful complementary metrics to evaluate MD ensembles.

### MZF1 cancer-related residues are hubs in the protein structure network and mediate long-range communication

A protein structure network (PSN) approach (Di Paola et al., 2013; Papaleo, 2015; Papaleo et al., 2016) was applied in the analyses of MZF1 molecular dynamics, as detailed in the Materials and Methods Section. The PSN method employs the graph formalism to identify a network of interacting residues in a given protein from the number of non-covalent contacts in the protein.

Two main properties of a PSN are the hub residues, i.e., residues that are highly connected within the network and the connected components, i.e., clusters of residues which are inter-connected but do not interact with residues in other clusters.

We evaluated the convergence of these two properties in our MD simulations by comparing their distribution in the average PSN and in PSNs generated by the shortest subsets of the trajectories (see Section Materials and Methods; Figures S4, S5). The analyses were carried out using two different distance cutoffs (0.5 and 0.55 nm) for PSN edge definition. We observed that if the distance cutoff is higher than 0.55 nm most of the protein residues are grouped in the same connected component (Figure S4), suggesting that this value is too high to achieve a proper network description of the system and that 0.5 nm is a suitable cutoff for the PSN analyses of the MZF1 MD ensembles. The result is force field independent since we observed the same behavior in all the MD simulations, i.e., a loss in the number of elements in the connected components with indexes higher than one.

**Figure 9:**
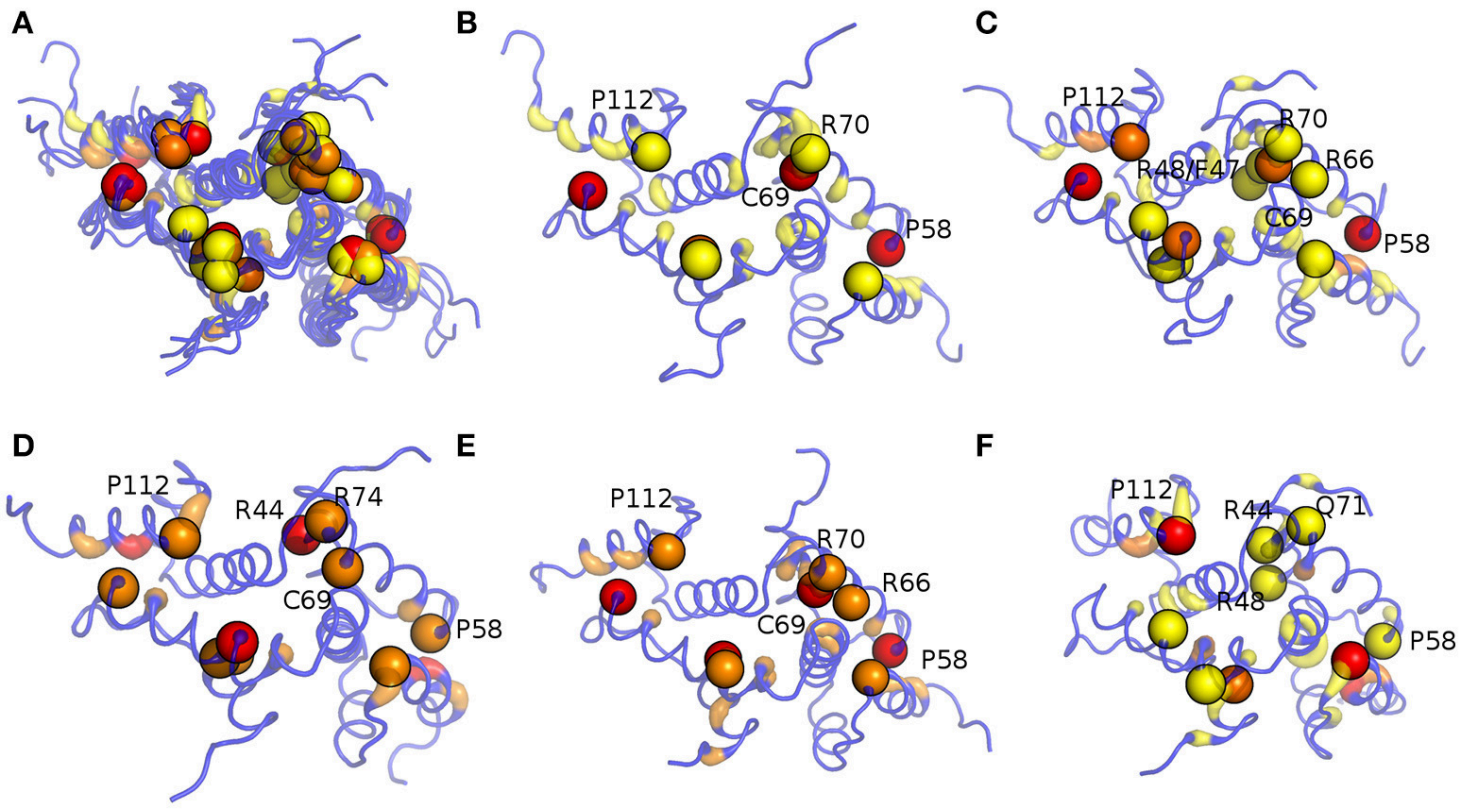
**Hub localization on MZF1 dimeric structure upon PSN analyses of the MD simulations**. Since in a PSN a hub is defined as a residue connected by at least three edges, all the residues with a degree lower than three are set at zero. The structure is depicted as ribbon with rainbow shades of colors from blue to red according to the node degree. The MZF1 residues for which mutations have been collected from different databases reported in Table S2 are depicted as spheres centered on their Cα atoms. The results for all the MD simulations **(A)**, Amber99-SB-NMR-ILDN **(B)**, Amber99-SB^*^-ILDN **(C)**, CHARMM22^*^ **(D)**, CHARMM27 **(E)**, and RSFF1 **(F)** simulations are shown in the different panels.

The connection degree of the hub residues in MZF1 is not higher than five in all the simulations and the hub distribution profiles are also independent on the force field used for the simulations, especially when force fields of the same family are compared (Figure S5), such as AMBER or CHARMM. Hubs in the networks have the important role of shortening the “communication” between distal nodes, and thus, they can play a crucial role in mediating structural effects over long distances in the protein structure. Furthermore, hubs preserve robustness of the network. Changes in the small degree nodes should not have a marked effect on the network integrity whereas, if important hubs are affected (especially the largest one), the network integrity can be easily compromised. We therefore evaluated if each of the mutation sites investigated so far constitutes a hub in the MD-derived PSNs of MZF1 (Figure 9). The MZF1 hub residues are generally located at the interface between the two monomers, highlighting the importance of the dimer architecture for the function of SCAN domains. Moreover, MZF1 hub residues are enriched in arginine or proline residues and mutation sites such as P112, P58, C69, R44, R74, and F47 have been identified as hub residues in the MZF1 simulations and are among the mutations that are predicted to affect the monomer stability or the protein-protein interface (Figure 5E). Of these residues, P112, P58, and C69 are not only the residues with more deleterious effects in terms of free energy of binding and stability but also hubs with the highest connection degree in the PSN.

**Figure 10:**
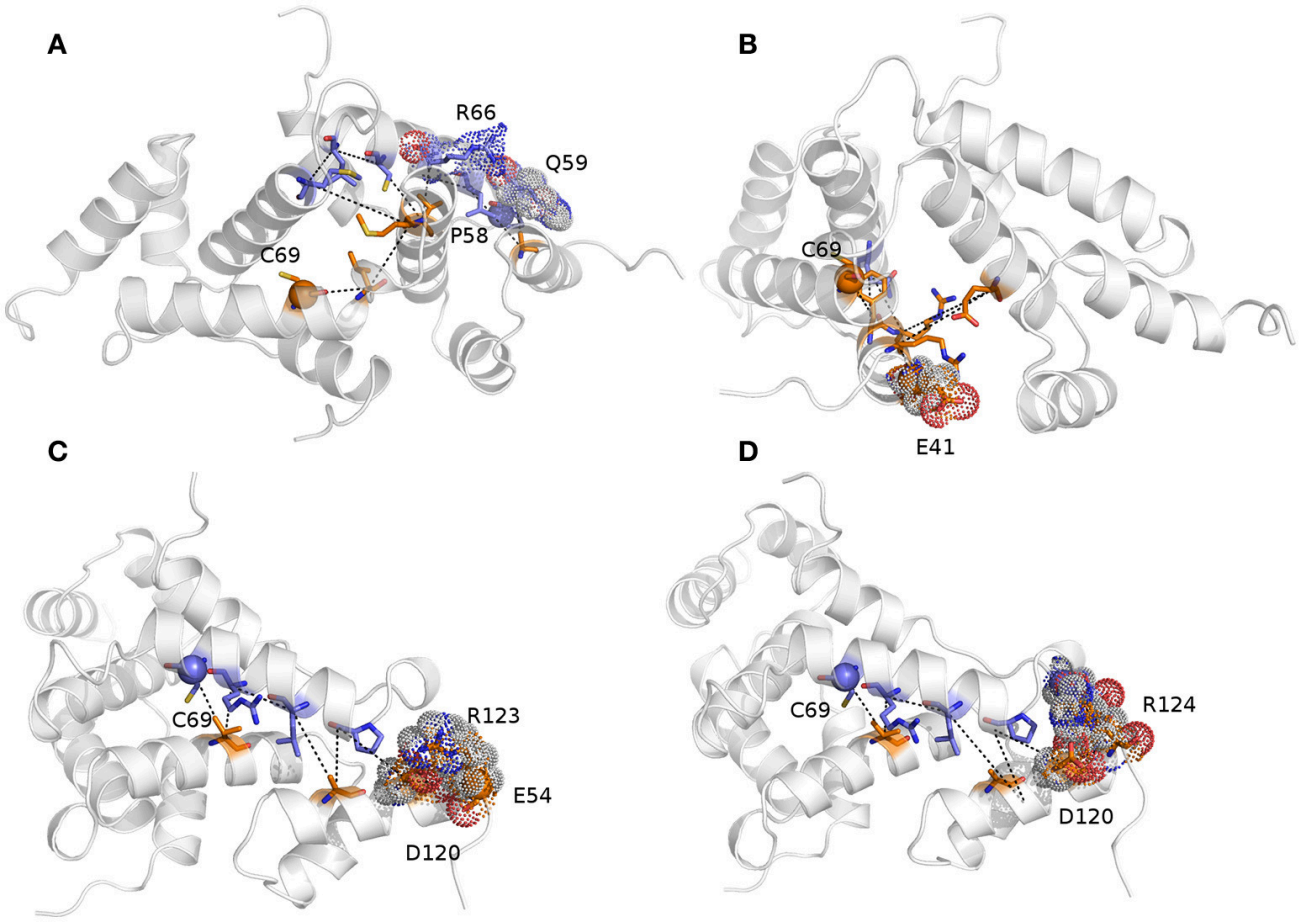
**The shortest paths of communications between C69 and P58 (A)**, E41 **(B)**, E54 **(C)**, and R124 **(D)** are shown.The initial and terminal residues of each path are highlighted as spheres. Chain A and B are colored in orange and blue, respectively. The intermediate nodes in each path are shown as sticks and the residues on the protein surface discussed in the text are shown as dots. The blue, red, and gray colors refer to the different atom types (i.e., N, O, and C, respectively). The paths are the following: C69_A_-> V88_A_->R66_B_->L62_B_->A116_A_->Q59_B_->P58_B_ (sum of weights 254.2, average weight 42.4); C69_A_->F47_A_->Q91_B_->R48_A_->E90_A_->R44_A_->E41_A_ (sum of weights 411.6, average weight 68.6); 69C_B_->88V_A_->66R_B_->62L_B_->A116_A_->P58_B_->D120_A_->R123_A_->E54_A_ (sum of weights 372.7, average weight 46.6); and C69_B_ ->V88_A_->R66_B_->L62_B_->A116_A_->P58_B_->D120_A_->R124_A_ (sum of weights 336.1, average weight 48).

We then calculated the communication paths between the MZF1 mutation sites and the interface for SCAN dimerization in the CHARMM27 simulation that is the MD ensemble associated to the highest predicted structural resolution (Figure 8). We selected the residues (Figures 5C,D) that encompass the highest impact on binding free energies (Y52, P58, L62, C69, L84, L87, and A95) as interface residues for the path calculation according to the saturation mutagenesis scan *in silico*. The mutation sites involved in distal communication to the SCAN dimerization interface are mainly proline (P58, P112, and P97) and arginine sites (R124, R44, R66, and R70) along with C69. C69 is central in the MZF1 network and has a key role in the path communications over long distances since it allows the SCAN dimerization region to mediate effects over long distances to solvent exposed sites, which might work as recruitment point for other cofactors (Figure 10). Indeed, C69 allows the dimerization interface to communicate to three different surface hotspots where R66 (Figure 10A), E41 (Figure 10B), and a cluster of charged residues (E54, D120, R123, and R124, Figures 10C,D) are located. The shortest paths that transmit structural effects from the SCAN dimerization interface to these surface hotspots are involving inter-molecular contacts between the two monomers emphasizing the importance of a properly folded dimeric SCAN domain for MZF1 functionality. The intermediate or the end nodes of the paths mediated by C69 are also known mutation sites of MZF1, such as R48 which is mutated in head and neck cancer, E41 in bladder cancer and F47 that is mutated in different cancer types.

## Concluding remarks

We integrated a plethora of different computational methods to unveil the role of MZF1 alterations in different cancer types. In particular, we focused on the SCAN domain, which is an important building block for protein-protein interaction in transcription factors. A focus on SCAN domains in cancer is of notable interest if we consider that both SCAN-only regulatory proteins and a SCAN-only short isoform of *mzf1* with disordered N- and C-terminal tails are known to play major roles in cancer biology. The SCAN domain of MZF1, and SCAN domains in general, can serve as a powerful combinatorial network for regulation of gene expression since they can provide a great diversity in the signaling thanks to their heterodimeric complexes, complexes with other signaling proteins and further regulation by post-translational modifications as Cdk4-mediated phosphorylation predicted here.

Our results show a complex and heterogeneous role of MZF1 and its interaction network in cancer. The alterations- and mutational landscape of MZF1 is strongly cancer-type dependent. We observed a marked variability in the levels of mzf1 among patients with same cancer types. The same observation holds for several of the MZF1 interactors. Our results highlight the need for more studies on MZF1 alterations in cancer with a specific focus on different cancer subtypes and other available clinical data. The integration of information available about MZF1 biological partners in the same cancer studies is also crucial since these proteins can exert marked effects, contributing to reshape and modulate the MZF1-mediated effects in the cell.

Our work provides a computational framework that allows to bridge global changes, such as changes in the expression levels or mutations of a gene and its interactors, to a detailed atomistic and structural understanding of the effects induced by these alterations. Moreover, since we performed the mutation scan at a high-throughput level, we had the possibility to assess the impact of the cancer mutations and of any other possible amino acidic substitutions. These data sets provide a valuable source of information to predict the impact of MZF1 mutations that will be identified in future studies related to disease. The most deleterious cancer-mutations are in hotspots where all mutations would not be tolerated suggesting that any modifications at these sites could harbor a pathogenic effect. In a broader context, this also suggests that other similarly critical hotspots (where no mutations are tolerated) are likely candidates for cancer-related mutations and structural analyses. The ones presented here are a valuable addition to the molecular characterization of the mutational landscape of oncogenes or tumor suppressors. We could also identify a subset of cancer passenger mutations, i.e., mutations found in cancer patients but unlikely to have any major impact on cancer pathways since these have neutral effects on structure, interactions and protein dynamics.

However, caution has to be taken since the energy function employed here can only predict local effects and will neglect all the long-range contributions that can perturb in a cascade of conformational changes to very distal sites. To overcome this issue, the integration of experimentally-validated molecular dynamics, to better account for conformational heterogeneity of the protein, and graph analyses, to identify paths of communication between distal sites, can help to achieve a more complete description of the intricate structural mutational landscape of cancer-related proteins.

In particular, our study pointed out a major role of a cysteine residue (C69) at the biological interface for protein-protein interactions mediated by the SCAN domain of MZF1. Alterations at this site have only been reported in cancer cell lines so far, but our predictions suggest that it can be a critical disease-related hotspot. Indeed, it is at the cross-road between multiple key paths of communication from the biological interface for protein-protein interactions to other hotspots on the MZF1 surface which can act as recruitment sites for other biological partners.

## Author contributions

Conceived and designed the experiments: EP. Performed the experiments: MN, TT, VS, FR, MD, MV, ML. Analyzed the data: MN, TT, ML, EP. Discussion of the data: EP, MN, TT. Contributed reagents/materials/analysis tools: MN, TT, AV, VS, JS, SB, MT, EP. Wrote the paper: MN, TT, ML, MT, MJ, TK, EP.

### Conflict of interest statement

The authors declare that the research was conducted in the absence of any commercial or financial relationships that could be construed as a potential conflict of interest.

## References

[B1] Adzhubei I. Jordan D. M. Sunyaev S. R. (2013). Predicting functional effect of human missense mutations using PolyPhen-2. Curr. Protoc. Hum. Genet. 76:7.20, 7.20.1–7.20.41. 10.1002/0471142905.hg0720s76 PMC4480630 23315928

[B2] Ahmed J. Meinel T. Dunkel M. Murgueitio M. S. Adams R. Blasse C.. (2011). CancerResource: a comprehensive database of cancer-relevant proteins and compound interactions supported by experimental knowledge. Nucleic Acids Res. 39, 1–8. 10.1093/nar/gkq910 20952398 PMC3013779

[B3] Auton A. Abecasis G. R. Altshuler D. M. Durbin R. M. Bentley D. R. Chakravarti A.. (2015). A global reference for human genetic variation. Nature 526, 68–74. 10.1038/nature15393 26432245 PMC4750478

[B4] Baspinar A. Cukuroglu E. Nussinov R. Keskin O. Gursoy A. (2014). PRISM: a web server and repository for prediction of protein-protein interactions and modeling their 3D complexes. Nucleic Acids Res. 42, W285–W289. 10.1093/nar/gku397 24829450 PMC4086120

[B5] Beauchamp K. A. Lin Y.-S. Das R. Pande V. S. (2012). Are Protein force fields getting better? a systematic benchmark on 524 diverse NMR measurements. J. Chem. Theory Comput. 8, 1409–1414. 10.1021/ct2007814 22754404 PMC3383641

[B6] Berjanskii M. Zhou J. Liang Y. Lin G. Wishart D. S. (2012). Resolution-by-proxy: a simple measure for assessing and comparing the overall quality of NMR protein structures. J. Biomol. NMR 53, 167–180. 10.1007/s10858-012-9637-2 22678091

[B7] Bernardi R. Pandolfi P. P. (2007). Structure, dynamics and functions of promyelocytic leukaemia nuclear bodies. Nat. Rev. Mol. Cell Biol. 8, 1007–1016. 10.1038/nrm2277 17928811

[B8] Best R. B. Hummer G. (2009). Optimized molecular dynamics force fields applied to the helix-coil transition of polypeptides. J. Phys. Chem. B 113, 9004–9015. 10.1021/jp901540t 19514729 PMC3115786

[B9] Best R. B. Zhu X. Shim J. Lopes P. E. M. Mittal J. Feig M. (2012). Optimization of the additive CHARMM all-atom protein force field targeting improved sampling of the backbone Φ, ψ and side-chain χ1 and χ2 dihedral angles. 8, 3257–3273. 10.1021/ct300400x PMC3549273 23341755

[B10] Bjelkmar P. Larsson P. Cuendet M. A. Hess B. Lindahl E. (2010). Implementation of the CHARMM force field in GROMACS: analysis of protein stability effects from correction maps, virtual interaction sites, and water models. J. Chem. Theory Comput. 6, 459–466. 10.1021/ct900549r 26617301

[B11] Bromberg Y. Yachdav G. Rost B. (2008). SNAP predicts effect of mutations on protein function. Bioinformatics 24, 2397–2398. 10.1093/bioinformatics/btn435 18757876 PMC2562009

[B12] Bussi G. Donadio D. Parrinello M. (2007). Canonical sampling through velocity rescaling. J. Chem. Phys. 126, 14101. 10.1063/1.2408420 17212484

[B13] Case D. A. (2013). Chemical shifts in biomolecules. Curr. Opin. Struct. Biol. 23, 172–179. 10.1016/j.sbi.2013.01.007 23422068 PMC3877577

[B14] Cerami E. Gao J. Dogrusoz U. Gross B. E. Sumer S. O. Aksoy B. A.. (2012). The cBio cancer genomics portal: an open platform for exploring multidimensional cancer genomics data. Cancer Discov. 2, 401–404. 10.1158/2159-8290.CD-12-0095 22588877 PMC3956037

[B15] Chen P. M. Cheng Y. W. Wang Y. C. Wu T. C. Chen C. Y. Lee H. (2014). Up-regulation of FOXM1 by E6 oncoprotein through the MZF1/NKX2-1 axis is required for human papillomavirus-associated tumorigenesis. Neoplasia (United States) 16, 961–971. 10.1016/j.neo.2014.09.010 25425970 PMC4240922

[B16] Choi Y. Sims G. E. Murphy S. Miller J. R. Chan A. P. (2012). Predicting the functional effect of amino acid substitutions and indels. PLoS ONE 7:e46688. 10.1371/journal.pone.0046688 23056405 PMC3466303

[B17] Colaprico A. Silva T. C. Olsen C. Garofano L. Cava C. Garolini D.. (2015). TCGAbiolinks: an R/Bioconductor package for integrative analysis of TCGA data. Nucleic Acids Res. 44:gkv1507. 10.1093/nar/gkv1507 26704973 PMC4856967

[B18] Di Paola L. De Ruvo M. Paci P. Santoni D. Giuliani A. (2013). Protein contact networks: an emerging paradigm in chemistry. Chem. Rev. 113, 1598–1613. 10.1021/cr3002356 23186336

[B19] Dong X.-Y. Yang X. Wang Y.-D. Chen W.-F. (2004). Zinc-finger protein ZNF165 is a novel cancer-testis antigen capable of eliciting antibody response in hepatocellular carcinoma patients. Br. J. Cancer 91, 1566–1570. 10.1038/sj.bjc.6602138 15354214 PMC2409927

[B20] Dror R. O. Dirks R. M. Grossman J. P. Xu H. Shaw D. E. (2012). Biomolecular simulation: a computational microscope for molecular biology. Annu. Rev. Biophys. 41, 429–452. 10.1146/annurev-biophys-042910-155245 22577825

[B21] Edelstein L. C. Collins T. (2005). The SCAN domain family of zinc finger transcription factors. Gene 359, 1–17. 10.1016/j.gene.2005.06.022 16139965

[B22] Eguchi T. Prince T. Wegiel B. Calderwood S. K. (2015). Role and regulation of myeloid zinc finger protein 1 in cancer. J. Cell. Biochem. 116, 2146–2154. 10.1002/jcb.25203 25903835 PMC7380561

[B23] Essmann U. Perera L. Berkowitz M. L. Darden T. Lee H. Pedersen L. G. (1995). A smooth particle mesh Ewald method. J. Chem. Phys. 103, 8577 10.1063/1.470117

[B24] Fersht A. R. Serrano L. (1993). Principles of protein stability derived from protein engineering experiments. Curr. Opin. Struct. Biol. 3, 75–83. 10.1016/0959-440X(93)90205-Y

[B25] Finn R. D. Coggill P. Eberhardt R. Y. Eddy S. R. Mistry J. Mitchell A. L.. (2016). The Pfam protein families database: towards a more sustainable future. Nucleic Acids Res. 44, D279–D285. 10.1093/nar/gkv1344 26673716 PMC4702930

[B26] Forbes S. A. Beare D. Gunasekaran P. Leung K. Bindal N. Boutselakis H.. (2015). COSMIC: exploring the world's knowledge of somatic mutations in human cancer. Nucleic Acids Res. 43, D805–D811. 10.1093/nar/gku1075 25355519 PMC4383913

[B27] Friendly M. (2002). Corrgrams: exploratory displays for correlation matrices. Am. Stat. 56, 316–324. 10.1198/000313002533

[B28] Fröhlich H. Speer N. Poustka A. Beissbarth T. (2007). GOSim–an R-package for computation of information theoretic GO similarities between terms and gene products. BMC Bioinformatics 8:166. 10.1186/1471-2105-8-166 17519018 PMC1892785

[B29] Gaboli M. Kotsi P. A. Gurrieri C. Cattoretti G. Ronchetti S. Broxmeyer H. E.. (2001). Mzf1 controls cell proliferation and tumorigenesis service Mzf1 controls cell proliferation and tumorigenesis. Genes Dev. 15, 1625–1630. 10.1101/gad.902301 11445537 PMC312729

[B30] Guerois R. Nielsen J. E. Serrano L. (2002). Predicting changes in the stability of proteins and protein complexes: a study of more than 1000 mutations. J. Mol. Biol. 320, 369–387. 10.1016/S0022-2836(02)00442-4 12079393

[B31] Guvench O. MacKerell A. D. (2008). Comparison of protein force fields for molecular dynamics simulations. 443, 63–88. 10.1007/978-1-59745-177-2_4 18446282

[B32] Henriques J. Cragnell C. Skepö M. (2015). Molecular dynamics simulations of intrinsically disordered proteins: force field evaluation and comparison with experiment. J. Chem. Theory Comput. 11, 3420–3431. 10.1021/ct501178z 26575776

[B33] Hess B. (2002). Convergence of sampling in protein simulations. Phys. Rev. E Stat. Nonlin. Soft Matter Phys. 65:31910. 10.1103/PhysRevE.65.031910 11909112

[B34] Hess B. Bekker H. Berendsen H. Fraaije J. (1993). LINCS: a linear constraint solver for molecular simulations. J. Comput. Chem. 12, 1463–1472.

[B35] Hess B. Kutzner C. van der Spoel D. Lindahl E. (2008). GROMACS 4: algorithms for highly efficient, load-balanced, and scalable molecular simulation. J. Chem. Theory Comput. 4, 435–447. 10.1021/ct700301q 26620784

[B36] Horn H. W. Swope W. C. Pitera J. W. Madura J. D. Dick T. J. Hura G. L.. (2004). Development of an improved four-site water model for biomolecular simulations: TIP4P-Ew. J. Chem. Phys. 120, 9665–9678. 10.1063/1.1683075 15267980

[B37] Hromas R. Davis B. Rauscher F. J. Klemsz M. Tenen D. Hoffman S.. (1996). Hematopoietic transcriptional regulation by the myeloid zinc finger gene, MZF-1. Curr. Top. Microbiol. Immunol. 211, 159–64. 10.1007/978-3-642-85232-9_16 8585946

[B38] Hsieh Y.-H. Wu T.-T. Huang C.-Y. Hsieh Y.-S. Liu J.-Y. (2007). Suppression of tumorigenicity of human hepatocellular carcinoma cells by antisense oligonucleotide MZF-1. Chin. J. Physiol. 50, 9–15. 17593797

[B39] Huang P.-J. Lee C.-C. Tan B. C.-M. Yeh Y.-M. Julie Chu L. Chen T.-W.. (2015). CMPD: cancer mutant proteome database. Nucleic Acids Res. 43, D849–D855. 10.1093/nar/gku1182 25398898 PMC4383976

[B40] Hudson T. J. Anderson W. Artez A. Barker A. D. Bell C. Bernabé R. R.. (2010). International network of cancer genome projects. Nature 464, 993–998. 10.1038/nature08987 20393554 PMC2902243

[B41] Invernizzi G. Tiberti M. Lambrughi M. Lindorff-Larsen K. Papaleo E. (2014). Communication routes in ARID domains between distal residues in helix 5 and the DNA-binding loops. PLoS Comput. Biol. 10:e1003744. 10.1371/journal.pcbi.1003744 25187961 PMC4154638

[B42] Jiang F. Zhou C.-Y. Wu Y.-D. (2014). Residue-specific force field based on the protein coil library. RSFF1: modification of OPLS-AA/L. J. Phys. Chem. B 118, 6983–6998. 10.1021/jp5017449 24815738

[B43] Johnson W. E. Li C. Rabinovic A. (2007). Adjusting batch effects in microarray expression data using empirical Bayes methods. Biostatistics 8, 118–127. 10.1093/biostatistics/kxj037 16632515

[B44] Jónsdóttir L. B. Ellertsson B. Invernizzi G. Magnúsdóttir M. Thorbjarnardóttir S. H. Papaleo E.. (2014). The role of salt bridges on the temperature adaptation of aqualysin I, a thermostable subtilisin-like proteinase. Biochim. Biophys. Acta Prot. Proteomics 1844, 2174–2181. 10.1016/j.bbapap.2014.08.011 25172393

[B45] Kotlyar M. Pastrello C. Sheahan N. Jurisica I. (2016). Integrated interactions database: tissue-specific view of the human and model organism interactomes. Nucleic Acids Res. 44, D536–D541. 10.1093/nar/gkv1115 26516188 PMC4702811

[B46] Kumar M. D. S. (2006). ProTherm and ProNIT: thermodynamic databases for proteins and protein-nucleic acid interactions. Nucleic Acids Res. 34, D204–D206. 10.1093/nar/gkj103 16381846 PMC1347465

[B47] Lambrughi M. De Gioia L. Gervasio F. L. Lindorff-Larsen K. Nussinov R. Urani C.. (2016a). DNA-binding protects p53 from interactions with cofactors involved in transcription-independent functions. Nucleic Acids Res. 44:gkw770. 10.1093/nar/gkw770 27604871 PMC5100575

[B48] Lambrughi M. Lucchini M. Pignataro M. Sola M. Bortolotti C. A. Gupta V. A. (2016b). The dynamics of the β-propeller domain in Kelch protein KLHL40 changes upon nemaline myopathy-associated mutation. RSC Adv. 6, 34043–34054. 10.1039/C6RA06312H

[B49] Lange O. F. van der Spoel D. de Groot B. L. (2010). Scrutinizing molecular mechanics force fields on the submicrosecond timescale with NMR data. Biophys. J. 99, 647–655. 10.1016/j.bpj.2010.04.062 20643085 PMC2905107

[B50] Lee S. Li S. Seo C. H. Lim B. Yang J. O. Oh J.. (2011). Accurate quantification of transcriptome from RNA-Seq data by effective length normalization. Nucleic Acids Res. 39:e9. 10.1093/nar/gkq1015 21059678 PMC3025570

[B51] Leek J. T. Johnson W. E. Parker H. S. Jaffe A. E. Storey J. D. (2012). The SVA package for removing batch effects and other unwanted variation in high-throughput experiments. Bioinformatics 28, 882–883. 10.1093/bioinformatics/bts034 22257669 PMC3307112

[B52] Li B. Krishnan V. G. Mort M. E. Xin F. Kamati K. K. Cooper D. N.. (2009). Automated inference of molecular mechanisms of disease from amino acid substitutions. Bioinformatics 25, 2744–2750. 10.1093/bioinformatics/btp528 19734154 PMC3140805

[B53] Li D. Brüschweiler R. (2015). PPM_One: a static protein structure based chemical shift predictor. J. Biomol. NMR 62, 403–409. 10.1007/s10858-015-9958-z 26091586

[B54] Li D. W. Brüschweiler R. (2010). NMR-based protein potentials. Angew. Chem. Int. Ed. 49, 6778–6780. 10.1002/anie.201001898 20715028

[B55] Li J. Duncan D. T. Zhang B. (2010). CanProVar: a human cancer proteome variation database. Hum. Mutat. 31, 219–228. 10.1002/humu.21176 20052754 PMC2829365

[B56] Liang Y. Huimei Hong F. Ganesan P. Jiang S. Jauch R. Stanton L. W.. (2012). Structural analysis and dimerization profile of the SCAN domain of the pluripotency factor Zfp206. Nucleic Acids Res. 40, 8721–8732. 10.1093/nar/gks611 22735705 PMC3458555

[B57] Lindorff-Larsen K. Maragakis P. Piana S. Eastwood M. P. Dror R. O. Shaw D. E. (2012). Systematic validation of protein force fields against experimental data. PLoS ONE 7:e32131. 10.1371/journal.pone.0032131 22384157 PMC3285199

[B58] Lindorff-Larsen K. Piana S. Palmo K. Maragakis P. Klepeis J. L. Dror R. O.. (2010). Improved side-chain torsion potentials for the Amber ff99SB protein force field. Proteins 78, 1950–1958. 10.1002/prot.22711 20408171 PMC2970904

[B59] Lovell S. C. Word J. M. Richardson J. S. Richardson D. C. (2000). The penultimate rotamer library. Proteins 40, 389–408. 10.1002/1097-0134(20000815)40:3<389::AID-PROT50>3.0.CO;2-2 10861930

[B60] MacKerell A. D. Bashford D. Dunbrack R. L. Evanseck J. D. Field M. J. Fischer S.. (1998). All-atom empirical potential for molecular modeling and dynamics studies of proteins^†^. J. Phys. Chem. B 102, 3586–3616. 10.1021/jp973084f 24889800

[B61] Mackerell A. D. Feig M. Brooks C. L. (2004). Extending the treatment of backbone energetics in protein force fields: limitations of gas-phase quantum mechanics in reproducing protein conformational distributions in molecular dynamics simulations. J. Comput. Chem. 25, 1400–1415. 10.1002/jcc.20065 15185334

[B62] Martín-García F. Papaleo E. Gomez-Puertas P. Boomsma W. Lindorff-Larsen K. (2015). Comparing molecular dynamics force fields in the essential subspace. PLoS ONE 10:e0121114. 10.1371/journal.pone.0121114 25811178 PMC4374674

[B63] Mashiach E. Nussinov R. Wolfson H. J. (2010). FiberDock: Flexible induced-fit backbone refinement in molecular docking. Proteins Struct. Funct. Bioinformatics 78, 1503–1519. 10.1002/prot.22668 20077569 PMC4290165

[B64] Mathe E. Olivier M. Kato S. Ishioka C. Hainaut P. Tavtigian S. V. (2006). Computational approaches for predicting the biological effect of p53 missense mutations: a comparison of three sequence analysis based methods. Nucleic Acids Res. 34, 1317–1325. 10.1093/nar/gkj518 16522644 PMC1390679

[B65] Mathiassen S. G. Larsen I. B. Poulsen E. G. Madsen C. T. Papaleo E. Lindorff-Larsen K.. (2015). A two-step protein quality control pathway for a misfolded DJ-1 variant in fission yeast. J. Biol. Chem. 290, 21141–21153. 10.1074/jbc.M115.662312 26152728 PMC4543670

[B66] Miller R. G. (1974). The jackknife-a review. Biometrika 61, 1–15.

[B67] Mitchell A. Chang H.-Y. Daugherty L. Fraser M. Hunter S. Lopez R.. (2014). The InterPro protein families database: the classification resource after 15 years. Nucleic Acids Res. 43, D213–D221. 10.1093/nar/gku1243 25428371 PMC4383996

[B68] Monaco C. Helmer Citterich M. Caprini E. Vorechovsky I. Russo G. Croce C. M.. (1998). Molecular cloning and characterization of ZNF202: a new gene at 11q23.3 encoding testis-specific zinc finger proteins. Genomics 52, 358–362. 10.1006/geno.1998.5419 9790754

[B69] Mudduluru G. Vajkoczy P. Allgayer H. (2010). Myeloid zinc finger 1 induces migration, invasion, and *in vivo* metastasis through Axl gene expression in solid cancer. Mol. Cancer Res. 8, 159–169. 10.1158/1541-7786.MCR-09-0326 20145042

[B70] Nan J. Jieyu W. Qing L. Xiang T. Kaikai C. Keqin H. (2016). DNA methylation promotes paired box 2 expression via myeloid zinc finger 1 in endometrial cancer. [Epub ahead of print]. 10.18632/oncotarget.12626 27764784 PMC5356698

[B71] Niroula A. Urolagin S. Vihinen M. (2015). PON-P2: prediction method for fast and reliable identification of harmful variants. PLoS ONE 10:e0117380. 10.1371/journal.pone.0117380 25647319 PMC4315405

[B72] Noll L. Peterson F. C. Hayes P. L. Volkman B. F. Sander T. (2008). Heterodimer formation of the myeloid zinc finger 1 SCAN domain and association with promyelocytic leukemia nuclear bodies. Leuk. Res. 32, 1582–1592. 10.1016/j.leukres.2008.03.024 18472161

[B73] Palmer A. G. (2015). Enzyme dynamics from NMR spectroscopy. Acc. Chem. Res. 48, 457–465. 10.1021/ar500340a 25574774 PMC4334254

[B74] Papaleo E. (2015). Integrating atomistic molecular dynamics simulations, experiments, and network analysis to study protein dynamics: strength in unity. Front. Mol. Biosci. 2:28. 10.3389/fmolb.2015.00028 26075210 PMC4445042

[B75] Papaleo E. Casiraghi N. Arrigoni A. Vanoni M. Coccetti P. De Gioia L. (2012a). Loop 7 of E2 enzymes : an ancestral conserved functional motif involved in the E2-mediated steps of the ubiquitination cascade. PLoS ONE 7:e40786. 10.1371/journal.pone.0040786 22815819 PMC3399832

[B76] Papaleo E. Parravicini F. Grandori R. De Gioia L. Brocca S. (2014a). Structural investigation of the cold-adapted acylaminoacyl peptidase from Sporosarcina psychrophila by atomistic simulations and biophysical methods. Biochim. Biophys. Acta 1844, 2203–2213. 10.1016/j.bbapap.2014.09.018 25280393

[B77] Papaleo E. Renzetti G. Tiberti M. (2012b). Mechanisms of intramolecular communication in a hyperthermophilic acylaminoacyl peptidase: a molecular dynamics investigation. PLoS ONE 7:e35686. 10.1371/journal.pone.0035686 22558199 PMC3338720

[B78] Papaleo E. Saladino G. Lambrughi M. Lindorff-Larsen K. Gervasio F. L. Nussinov R. (2016). The role of protein loops and linkers in conformational dynamics and allostery. Chem. Rev. 116, 6391–6423. 10.1021/acs.chemrev.5b00623 26889708

[B79] Papaleo E. Sutto L. Gervasio F. L. Lindorff-Larsen K. (2014b). Conformational changes and free energies in a proline isomerase. J. Chem. Theory Comput. 10, 4169–4174. 10.1021/ct500536r 26588555

[B80] Perrotti D. Melotti P. Skorski T. Casella I. Peschle C. Calabretta B. (1995). Overexpression of the zinc finger protein MZF1 inhibits hematopoietic development from embryonic stem cells: correlation with negative regulation of CD34 and c-myb promoter activity. Mol. Cell. Biol. 15, 6075–6087. 10.1128/MCB.15.11.6075 7565760 PMC230859

[B81] Peterson F. C. Hayes P. L. Waltner J. K. Heisner A. K. Jensen D. R. Sander T. L.. (2006). Structure of the SCAN domain from the tumor suppressor protein MZF1. J. Mol. Biol. 363, 137–147. 10.1016/j.jmb.2006.07.063 16950398 PMC1941711

[B82] Peterson M. J. Morris J. F. (2000). Human myeloid zinc finger gene MZF produces multiple transcripts and encodes a SCAN box protein. Gene 254, 105–118. 10.1016/S0378-1119(00)00281-X 10974541

[B83] Piana S. Klepeis J. L. Shaw D. E. (2014). Assessing the accuracy of physical models used in protein-folding simulations: quantitative evidence from long molecular dynamics simulations. Curr. Opin. Struct. Biol. 24, 98–105. 10.1016/j.sbi.2013.12.006 24463371

[B84] Piana S. Lindorff-Larsen K. Shaw D. E. (2011). How robust are protein folding simulations with respect to force field parameterization? Biophys. J. 100, L47–L49. 10.1016/j.bpj.2011.03.051 21539772 PMC3149239

[B85] Privalov P. L. (1979). Stability of proteins small globular proteins. Adv. Protein Chem. 33, 167–241. 10.1016/S0065-3233(08)60460-X 44431

[B86] Rafn B. Nielsen C. F. Andersen S. H. Szyniarowski P. Corcelle-Termeau E. Valo E.. (2012). ErbB2-driven breast cancer cell invasion depends on a complex signaling network activating myeloid zinc finger-1-dependent cathepsin B expression. Mol. Cell 45, 764–776. 10.1016/j.molcel.2012.01.029 22464443

[B87] Reimand J. Kull M. Peterson H. Hansen J. Vilo J. (2007). G:Profiler-a web-based toolset for functional profiling of gene lists from large-scale experiments. Nucleic Acids Res. 35, 193–200. 10.1093/nar/gkm226 17478515 PMC1933153

[B88] Reva B. Antipin Y. Sander C. (2011). Predicting the functional impact of protein mutations: application to cancer genomics. Nucleic Acids Res. 39, e118–e118. 10.1093/nar/gkr407 21727090 PMC3177186

[B89] Rimsa V. Eadsforth T. C. Hunter W. N. (2013). Structure of the SCAN domain of human paternally expressed gene 3 protein. PLoS ONE 8:e69538. 10.1371/journal.pone.0069538 23936039 PMC3720700

[B90] Risso D. Schwartz K. Sherlock G. Dudoit S. (2011). GC-content normalization for RNA-Seq data. BMC Bioinformatics 12:480. 10.1186/1471-2105-12-480 22177264 PMC3315510

[B91] Robustelli P. Stafford K. A. Palmer A. G. (2012). Interpreting protein structural dynamics from NMR chemical shifts. J. Am. Chem. Soc. 134, 6365–6374. 10.1021/ja300265w 22381384 PMC3324661

[B92] Sander T. L. Haas A. L. Peterson M. J. Morris J. F. (2000). Identification of a novel SCAN box-related protein that interacts with MZF1B. J. Biol. Chem. 275, 12857–12867. 10.1074/jbc.275.17.12857 10777584

[B93] Sander T. L. Stringer K. F. Maki J. L. Szauter P. Stone J. R. Collins T. (2003). The SCAN domain defines a large family of zinc finger transcription factors. Gene 310, 29–38. 10.1016/S0378-1119(03)00509-2 12801630

[B94] Schymkowitz J. Borg J. Stricher F. Nys R. Rousseau F. Serrano L. (2005). The FoldX web server: an online force field. Nucleic Acids Res. 33, W382–W388. 10.1093/nar/gki387 15980494 PMC1160148

[B95] Shannon P. Markiel A. Ozier O. Baliga N. S. Wang J. T. Ramage D.. (2003). Cytoscape: a software environment for integrated models of biomolecular interaction networks. Genome Res. 13, 2498–2504. 10.1101/gr.1239303 14597658 PMC403769

[B96] Sherry S. T. Ward M. H. Kholodov M. Baker J. Phan L. Smigielski E. M.. (2001). dbSNP: the NCBI database of genetic variation. Nucleic Acids Res. 29, 308–311. 10.1093/nar/29.1.308 11125122 PMC29783

[B97] Silva T. C. Colaprico A. Olsen C. D'Angelo F. Bontempi G. Ceccarelli M. (2016). TCGA workflow: analyze cancer genomics and epigenomics data using Bioconductor packages. F1000Research 5, 1542 10.12688/f1000research.8923.1 PMC5302158 28232861

[B98] Singh P. K. Srivastava A. K. Dalela D. Rath S. K. Goel M. M. Bhatt M. L. B. (2015). Frequent expression of zinc-finger protein ZNF165 in human urinary bladder transitional cell carcinoma. Immunobiology 220, 68–73. 10.1016/j.imbio.2014.08.018 25214475

[B99] Sudmeier J. L. Bradshaw E. M. Coffman Haddad K. E. Day R. M. Thalhauser C. J. Bullock P. A.. (2003). Identification of histidine tautomers in proteins by 2D 1H/13Cδ2 one-bond correlated NMR. J. Am. Chem. Soc. 125, 8430–8431. 10.1021/ja034072c 12848537

[B100] Tavtigian S. V. (2005). Comprehensive statistical study of 452 BRCA1 missense substitutions with classification of eight recurrent substitutions as neutral. J. Med. Genet. 43, 295–305. 10.1136/jmg.2005.033878 16014699 PMC2563222

[B101] Tiberti M. Invernizzi G. Lambrughi M. Inbar Y. Schreiber G. Papaleo E. (2014). PyInteraph : a framework for the analysis of interaction networks in structural ensembles of proteins. J. Chem. Inf. Model. 54, 1537–1551. 10.1021/ci400639r 24702124

[B102] Tiberti M. Invernizzi G. Papaleo E. (2015a). A (dis)similarity index to compare correlated motions in molecular simulations. J. Chem. Theory Comput. 11, 4404–4414. 10.1021/acs.jctc.5b00512 26575932

[B103] Tiberti M. Papaleo E. Bengtsen T. Boomsma W. Lindorff-Larsen K. (2015b). ENCORE: software for quantitative ensemble comparison. PLoS Comput. Biol. 11:e1004415. 10.1371/journal.pcbi.1004415 26505632 PMC4624683

[B104] Tokuriki N. Stricher F. Schymkowitz J. Serrano L. Tawfik D. S. (2007). The stability effects of protein mutations appear to be universally distributed. J. Mol. Biol. 369, 1318–1332. 10.1016/j.jmb.2007.03.069 17482644

[B105] Tomczak K. Czerwińska P. Wiznerowicz M. (2015). Review The Cancer Genome Atlas (TCGA): an immeasurable source of knowledge. Współczesna Onkol. 1A, 68–77. 10.5114/wo.2014.47136 PMC4322527 25691825

[B106] Tsai L.-H. Wu J.-Y. Cheng Y.-W. Chen C.-Y. Sheu G.-T. Wu T.-C.. (2015). The MZF1/c-MYC axis mediates lung adenocarcinoma progression caused by wild-type lkb1 loss. Oncogene 34, 1641–1649. 10.1038/onc.2014.118 24793789

[B107] Tuncbag N. Gursoy A. Nussinov R. Keskin O. (2011). Predicting protein-protein interactions on a proteome scale by matching evolutionary and structural similarities at interfaces using PRISM. Nat. Protoc. 6, 1341–1354. 10.1038/nprot.2011.367 21886100 PMC7384353

[B108] Unan H. Yildirim A. Tekpinar M. (2015). Opening mechanism of adenylate kinase can vary according to selected molecular dynamics force field. J. Comput. Aided Mol. Des. 29, 655–665. 10.1007/s10822-015-9849-0 26009297

[B109] Vishwamitra D. Curry C. V. Alkan S. Song Y. H. Gallick G. E. Kaseb A. O.. (2015). The transcription factors Ik-1 and MZF1 downregulate IGF-IR expression in NPM-ALK(+) T-cell lymphoma. Mol. Cancer 14, 324. 10.1186/s12943-015-0324-2 25884514 PMC4415347

[B110] Williams A. J. Blacklow S. C. Collins T. (1999). The zinc finger-associated SCAN box is a conserved oligomerization domain. Mol. Cell. Biol. 19, 8526–8535. 10.1128/MCB.19.12.8526 10567577 PMC84969

[B111] Williams A. J. Khachigian L. M. Shows T. Collins T. (1995). Isolation and characterization of a novel zinc-finger protein with transcription repressor activity. J. Biol. Chem. 270, 22143–22152. 10.1074/jbc.270.38.22143 7673192

[B112] Yang L. Wang H. Kornblau S. M. Graber D. A. Zhang N. Matthews J. A.. (2011). Evidence of a role for the novel zinc-finger transcription factor ZKSCAN3 in modulating Cyclin D2 expression in multiple myeloma. Oncogene 30, 1329–1340. 10.1038/onc.2010.515 21057542 PMC4128235

[B113] Yu G. Li F. Qin Y. Bo X. Wu Y. Wang S. (2010). GOSemSim: An R package for measuring semantic similarity among GO terms and gene products. Bioinformatics 26, 976–978. 10.1093/bioinformatics/btq064 20179076

