# Supplementary material for: The Mutational Landscape of the Oncogenic MZF1 SCAN Domain in Cancer
Source: Front Mol Biosci. 2016 Dec 15;3:78. doi: 10.3389/fmolb.2016.00078 (PMC5156680; doi:10.3389/fmolb.2016.00078)
Supplement: Supplementary file 1 [file Table1.DOCX]

**Table S1.** Summary of TCGA RNASEQ data set analyzed in our study. The significant differences between the mean of the distribution of tumor versus normal have been evaluated with a t-test and only differences with a p-value lower than 0.005 are here indicated.

| **ID** | **Cancer type** | **Primary site** | **Analyzed samples after preprocessing (all/paired)** | **Availability of matched paired samples** | **MZF1 expression levels in tumor compared to normal samples** |
| --- | --- | --- | --- | --- | --- |
| BLCA | Bladder Urothelial Carcinoma | Bladder | 465/38 | X | Higher |
| BRCA | Breast Invasive Carcinoma | Breast | 1439/228 | X | Higher |
| CESC | Cervical Squamous Cell Carcinoma and Endocervical Adenocarcinoma | Cervix | 314/6 | X (< 15) | Higher |
| CHOL | Cholangiocarcinoma | Bile Duct | 63/18 | X | Unchanged |
| COAD | Colon Adenocarcinoma | Colorectal | 379/52 | X | Higher |
| ESCA | Esophageal Carcinoma | Esophagus | 217/22 | X | Higher |
| GBM | Glioblastoma Multiforme | Brain | 161 | N.A. | Higher |
| HNSC | Head and Neck Squamous Cell Carcinoma | Head and Neck | 650/86 | X | Unchanged |
| KICH | Kidney Chromophobe | Kidney | 141/50 | X | Lower |
| KIRC | Kidney Renal Clear Cell Carcinoma | Kidney | 749/144 | X | Lower |
| KIRP | Kidney Renal Papillary Cell Carcinoma | Kidney | 386/64 | X | Higher |
| LIHC | Liver Hepatocellular Carcinoma | Liver | 521/100 | X | Unchanged |
| LUAD | Lung Adenocarcinoma | Lung | 690/116 | X | Higher |
| LUSC | Lung Squamous Cell Carcinoma | Lung | 612/102 | X | Higher |
| PAAD | Pancreatic Adenocarcinoma | Pancreas | 190/8 | X (< 15) | Higher |
| PCPG | Pheochromocytoma and paraganglioma | Adrenal Gland | 188/6 | X ( < 15) | Lower |
| PRAD | Prostate Adenocarcinoma | Prostate | 653/104 | X | Higher |
| READ | Rectum Adenocarcinoma | Colorectal | 116/12 | X | Higher |
| SARC | Sarcoma | Soft Tissue | 265/4 | X (< 15) | Unchanged |
| SKCM | Skin Cutaneous Melanoma | Skin | 104 | N.A. | Higher |
| STAD | Stomach Adenocarcinoma | Stomach | 514/64 | X | Higher |
| THCA | Thyroid Carcinoma | Thyroid | 682/118 | X | Unchanged |
| THYM | Thymoma | Thymus | 126/4 | X (< 15) | Higher |
| UCEC | Uterine Corpus Endometrial Carcinoma | Uterus | 548/14 | X (< 15) | Higher |
