# Supplementary material for: The Mutational Landscape of the Oncogenic MZF1 SCAN Domain in Cancer
Source: Front Mol Biosci. 2016 Dec 15;3:78. doi: 10.3389/fmolb.2016.00078 (PMC5156680; doi:10.3389/fmolb.2016.00078)
Supplement: Supplementary file 2 [file Table2.DOCX]

| **Uniprot ID** | **Gene Name** | **Description** | **Interaction database** | **Interaction experimental method** | **Domain Composition** |
| --- | --- | --- | --- | --- | --- |
| 075123 | ZNF623 | Zinc finger protein | Biogrid_Mouse | Two hybrid | ZF |
| 095125 | ZNF202 | Zinc finger protein | Biogrid | Reconstituted complex | SCAN, ZF |
| 095751 | LDOC1 | Leucine zipper | BCI, BIND, HPRD | In vitro – Two hybrid | / |
| P08571 | CD14 | CD14 molecule | INNATEDB | electrophoretic mobility  supershift assay | / |
| P0C7X2 | ZNF688 | Zinc finger protein | Biogrid_Mouse | Two hybrids | ZF |
| P11802 | CDK4 | Cyclin-dependent kinase 4 | Biogrid, IntAct | Biochemical activity | / |
| P17028 | ZNF24 | Zinc finger protein | Biogrid | Reconstituted complex | SCAN, ZF |
| P28698 | MZF1 | Myeloid Zinc Finger protein | Biogrid_Mouse  HPRD | Two hybrids  In vivo | SCAN, ZF |
| P57086 | SCAND1 | Scan domain containing 1 | Biogrid, HPRD | Affinity Capture-Western  Two hybrids  In vivo | SCAN |
| P63165 | SUMO1 | Small ubiquitin-like modifier 1 | Biogrid | N.A. | / |
| Q00534 | CDK6 | Cyclin-dependent kinase 6 | Biogrid, IntAct | Biochemical activity | / |
| Q13643 | FHL3 | LIM domain | INNATEDB, HPRD | In-vitro  Two hybrid  coimmunoprecipitation | / |
| Q13952 | NFYC | Nuclear transcription factor Y | INNATEDB | N.A. | / |
| Q15697 | ZNF174 | Zinc finger protein | Biogrid | Reconstituted complex | SCAN, ZF |
| Q7Z7L9 | ZSCAN2 | Zinc finger and SCAN domain containing 2 | Biogrid_Mouse | Two hybrids | SCAN, ZF |
| Q8N8E2 | ZNF513 | Zinc finger protein | Biogrid_Mouse | Two hybrids | ZF |
| Q8WTR7 | ZNF473 | Zinc finger protein | Biogrid_Mouse | Two hybrids | ZF |
| Q9H5H4 | ZNF768 | Zinc finger protein | Biogrid_Mouse | Two hybrids | ZF |

**Table S2**. Summary of MZF1 partners as derived by protein-protein interaction databases where experimentally identified interactions are annotated.
