# Supplementary material for: The Mutational Landscape of the Oncogenic MZF1 SCAN Domain in Cancer
Source: Front Mol Biosci. 2016 Dec 15;3:78. doi: 10.3389/fmolb.2016.00078 (PMC5156680; doi:10.3389/fmolb.2016.00078)
Supplement: Supplementary file 3 [file Table3.DOCX]

**Table S3. Cancer mutations in MZF1 SCAN domain.** The cancer-related mutations deposited in *Cbioportal*, *CMPD*, *ICGC Portal*, *CancerResource* and *COSMIC* and located in the known structure of the MZF1 SCAN domain (residue 35-128, PDB entry 2FI2) are here reported. Other MZF1 mutations, which have not been associated to disease yet, have been inferred by the analyses of *HumanSavar*, *CanProVar*, *1000 Genomes* and *dbSNP* and are reported in italic in the table. We also reported the predicted unfolding free energies of the mutant variants (∆Gu*_mutant_*) as explained in the Materials and Methods and Results in the main text. In the calculation the (∆Gu*_WT_* is assumed to be -5 kcal/mol. The associated value in parenthesis is the estimation of the error in the prediction which has been calculated estimating the ∆Gu of the corresponding self-mutation with *FoldX*. Indeed, the self-mutation should in principle give a ∆Gu value of -5 kcal/mol and allow us thus to have a further estimation of the uncertainty in the calculation.

| **Amino acidic substitution** | **Cancer Study (# mutations)** | **Source** | **∆Gu*_mutant_  ± error in self-mutation estimate*** |
| --- | --- | --- | --- |
| *P40S* | COSMIC (1) | COSMIC, dbSNP, 1000 Genomes | -2.88 ± 0.07 kcal/mol |
| E41K | BLCA-TCGA(1)  COSMIC (2)  ICGC (1) | Cbioportal, COSMIC, ICGC | -3.67 ± 0.06 kcal/mol |
| *R44G* | No disease association | dbSNP, 1000 Genomes | -3.21 ± 0.003 kcal/mol |
| *R44C* | ACC – TCGA (1) | Cbioportal, CMPD, dbSNP, 1000 Genomes | -4.00 ± 0.003 kcal/mol |
| R44H | COSMIC (1)  ICGC | COSMIC, ICGC | -3.31 ± 0.003 kcal/mol |
| L45M | NCI-60 CELL LINES (1)  COSMIC (1)  HCT-15 CELL LINES | Cbioportal, CMPD, COSMIC, CancerResource | -5.00 ± 0.007 kcal/mol |
| R46H | COLORECTAL – GENENTECH (1)  COSMIC (1) | Cbioportal, COSMIC | -4.33 ± 0.006 kcal/mol |
| F47S | PRCC-TCGA (1)  KIRP – TCGA (1)  COSMIC (1)  ICGC (1) | Cbioportal, CMPD, COSMIC, ICGC | -1.78 ± 0.006 kcal/mol |
| R48Q | HEAD AND NECK - BROAD (1)  COSMIC (1) | Cbioportal, COSMIC | -4.60 ± 0.006 kcal/mol |
| *R48L* | No disease association | dbSNP | -5.17 ± 0.006 kcal/mol |
| *R51H* | DLBC – TCGA (2)  NCL-60 CELL LINES (1)  ICGC (1) | Cbioportal,  CMPD, ICGC, HumanSavar, CanProVar, dbSNP, 1000 Genomes | -5.34 ± 0.008 kcal/mol |
| *R51C* | No disease association | dbSNP | -4.82 ± 0.008 kcal/mol |
| *Y52H* | No disease association | dbSNP | -3.91 ± 0.008 kcal/mol |
| *E54Q* | No disease association | dbSNP, 1000 Genomes | -4.60 ± 0.006 kcal/mol |
| G57W | COLORECTAL – GENENTECH (1)  COSMIC (1) | Cbioportal, COSMIC | -1.39 ± 0.007 kcal/mol |
| *P58L* | COSMIC (1) | COSMIC, dbSNP, 1000 Genomes | -4.71 ± 0.007 kcal/mol |
| *R66Q* | No disease association | dbSNP | -4.94 ± 0.007 kcal/mol |
| *C69Y* | TE-1, KYSE-520 Cell-lines | CancerResource, dbSNP, 1000 Genomes | -3.11 ± 0.007 kcal/mol |
| *R70H* | COSMIC (1)  ICGC (1) | COSMIC, ICGC, dbSNP, 1000 Genomes | -4.38 ± 0.006 kcal/mol |
| Q71R | STAD – TCGA (2)  COSMIC (1)  ICGC (1) | Cbioportal, CMPD, COSMIC, ICGC | -4.83 ± 0.006 kcal/mol |
| *R74C* | COSMIC (1) | COSMIC, dbSNP, 1000 Genomes | -3.34 ± 0.003 kcal/mol |
| *R78H* | No disease association | dbSNP | -4.35 ± 0.002 kcal/mol |
| *R78C* | No disease association | dbSNP, 1000 Genomes | -3.61 ± 0.002 kcal/mol |
| *S79P* | No disease association | dbSNP, 1000 Genomes | -5.20 ± 0.006 kcal/mol |
| *Q82R* | No disease association | dbSNP, 1000 Genomes | -4.68 ± 0.005 kcal/mol |
| L86V | NCI-H292 | CancerResource | -2.13 ± 0.005 kcal/mol |
| G94S | COLORECTAL – GENENTECH (1)  COSMIC (1) | Cbioportal  COSMIC | -5.23 ± 0.007 kcal/mol |
| *P97T* | No disease association | dbSNP | -3.14 ± 0.006 kcal/mol |
| E99Q | CESC – TCGA (1)  COSMIC (1)  ICGC (1) | Cbioportal, COSMIC, ICGC | -4.20 ± 0.005 kcal/mol |
| I100N | LNCaP-Clone-FGC CELL LINES | CancerResource | -2.04 ± 0.006 kcal/mol |
| A102T | COSMIC (1) | COSMIC | -4.80 ± 0.007 kcal/mol |
| *R103H* | No disease association | CanProVar, HumanSavar, dbSNP, 1000 Genomes | -4.52 ± 0.005 kcal/mol |
| *R103C* | No disease association | dbSNP | -3.97 ± 0.005 kcal/mol |
| *R103Q* | No disease association | CanProVar, HumanSavar | -4.67 ± 0.005 kcal/mol |
| *R108W* | No disease association | dbSNP,  1000 Genomes | -4.65 ± 0.002 kcal/mol |
| R108L | STAD – TCGA (2)  COSMIC (2)  ICGC (1) | Cbioportal, CMPD, COSMIC, ICGC | -5.08 ± 0.002 kcal/mol |
| *P112R* | No disease association | dbSNP | -2.56 ± 0.006 kcal/mol |
| *E113K* | No disease association | dbSNP, 1000 Genomes | -4.32 ± 0.006 kcal/mol |
| *R124W* | No disease association | dbSNP, 1000 Genomes | -4.01 ± 0.006 kcal/mol |
| *R124Q* | No disease association | dbSNP, 1000 Genomes | -4.27 ± 0.006 kcal/mol |
| E125K | NCI-H1563 | CancerResource | -5.13 ± 0.006 kcal/mol |
| *P126L* | COLORECTAL – GENENTECH (1) COSMIC (1) | Cbioportal, COSMIC, dbSNP | -4.37 ± 0.007 kcal/mol |
| *P126T* | No disease association | dbSNP | -4.14 ± 0.007 kcal/mol |
| *G127S* | No disease association | dbSNP | -3.80 ± 0.007 kcal/mol |
| *G128R* | No disease association | dbSNP, 1000 Genomes | -4.50 ± 0.007 kcal/mol |
