# Supplementary material for: The Mutational Landscape of the Oncogenic MZF1 SCAN Domain in Cancer
Source: Front Mol Biosci. 2016 Dec 15;3:78. doi: 10.3389/fmolb.2016.00078 (PMC5156680; doi:10.3389/fmolb.2016.00078)
Supplement: Supplementary file 4 [file Image1.PDF]

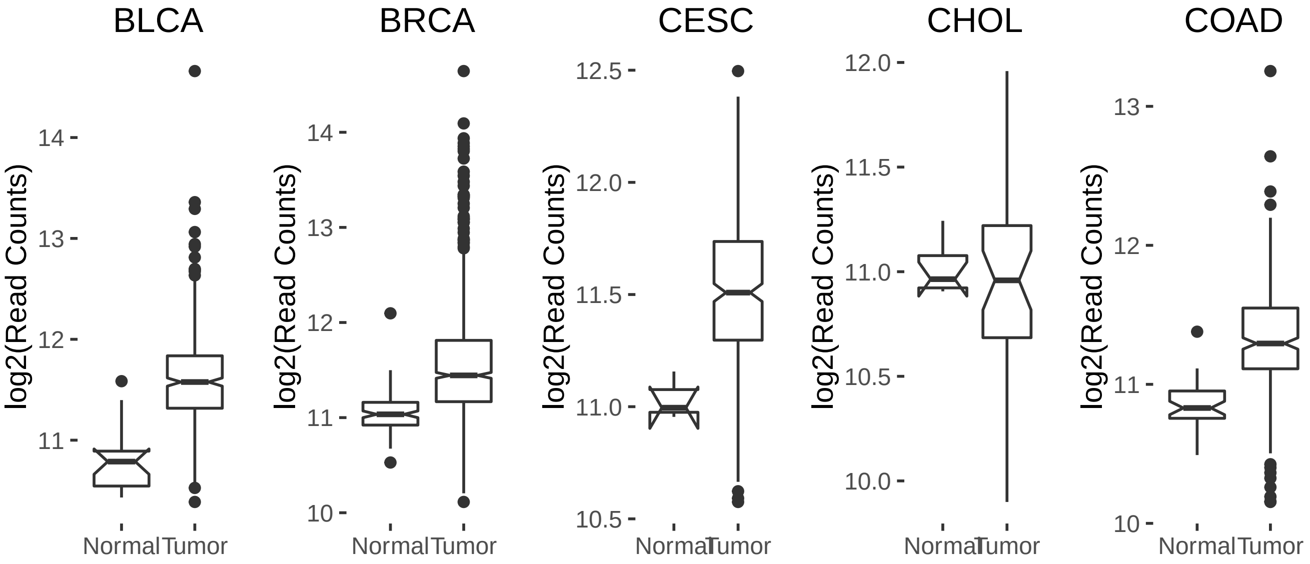

BLCA
BRCA
CESC
CHOL
COAD
log2(Read Counts)
14
13
12
11
10
12.5
12.0
11.5
11.0
10.5
Normal
Tumor

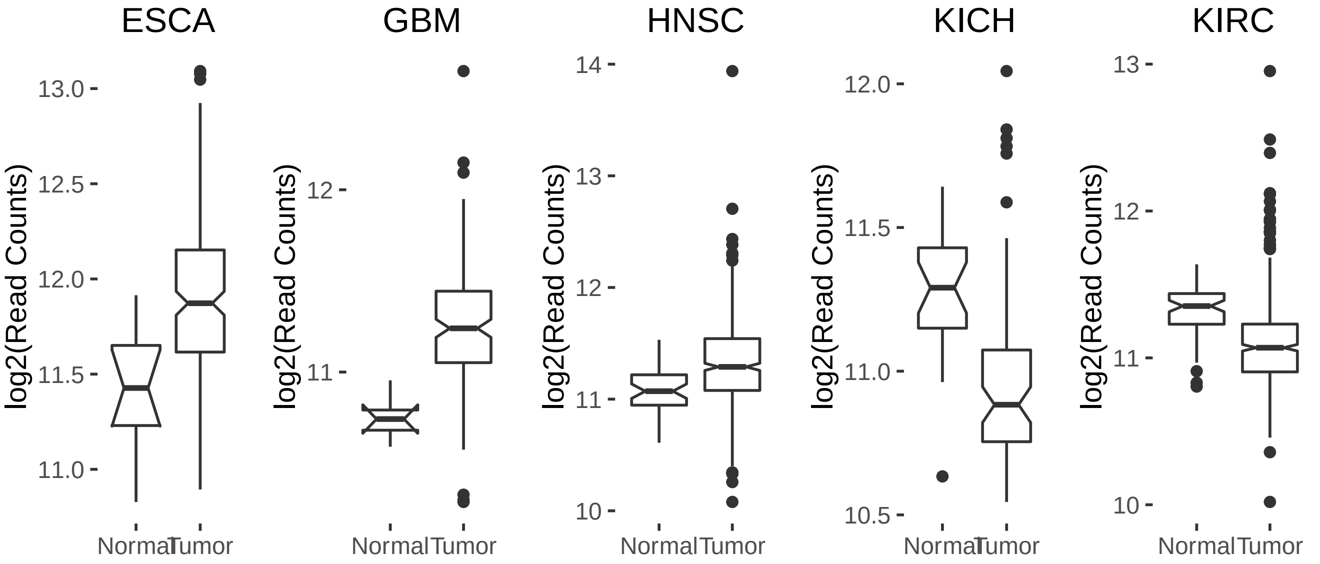

ESCA
GBM
HNSC
KICH
KIRC
log2(Read Counts)
13.0
12.5
12.0
11.5
11.0
12
11
14
13
12
11
10
12.0
11.5
11.0
10.5
13
12
11
10
Normal
Tumor

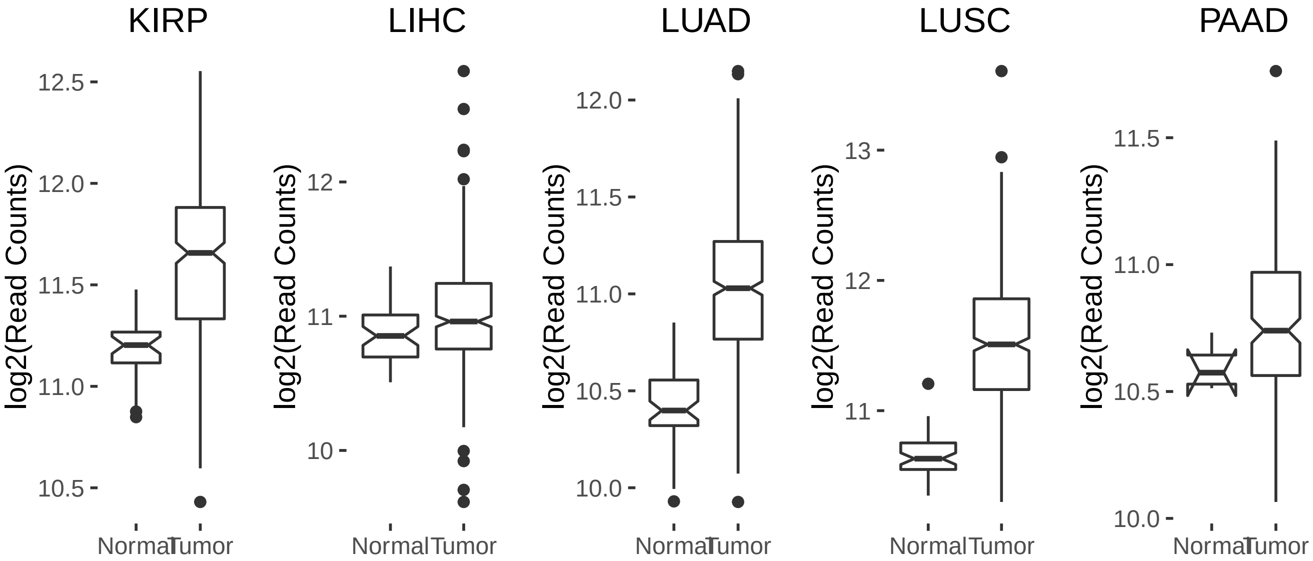
KIRP
LIHC
LUAD
LUSC
PAAD
log2(Read Counts)
12.5
12.0
11.5
11.0
10.5
12
11
10
10.0
13
Normal
Tumor

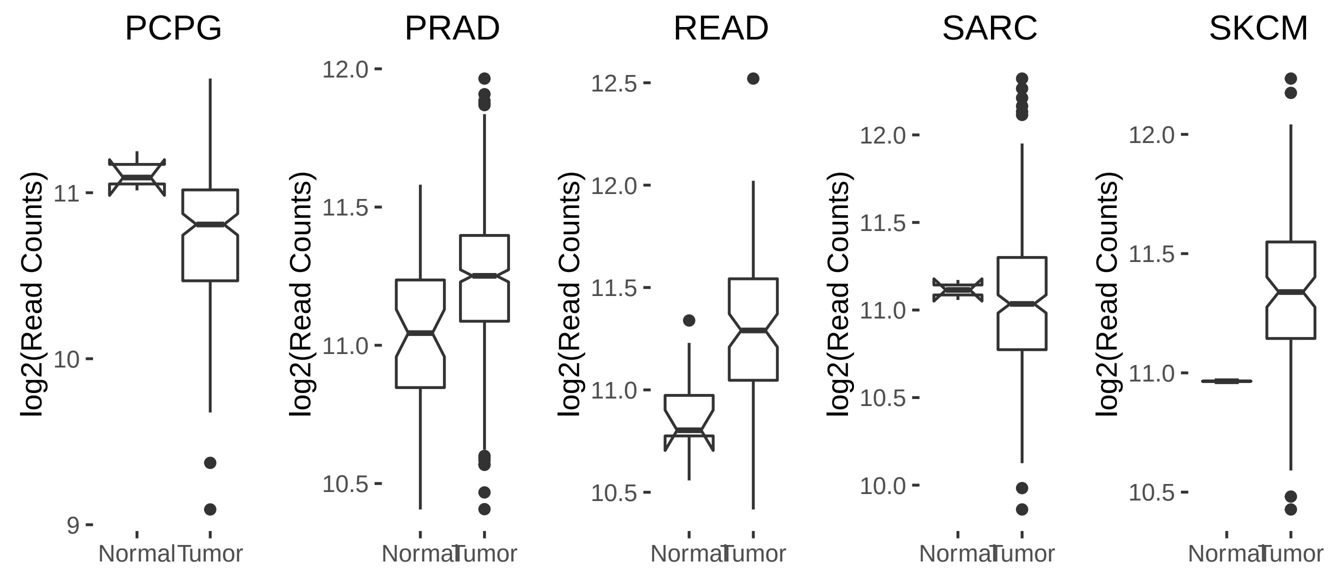

PCPG
PRAD
READ
SARC
SKCM
log2(Read Counts)
9
10
11
10.5
11.0
11.5
12.0
12.5
10.0
Normal
Tumor

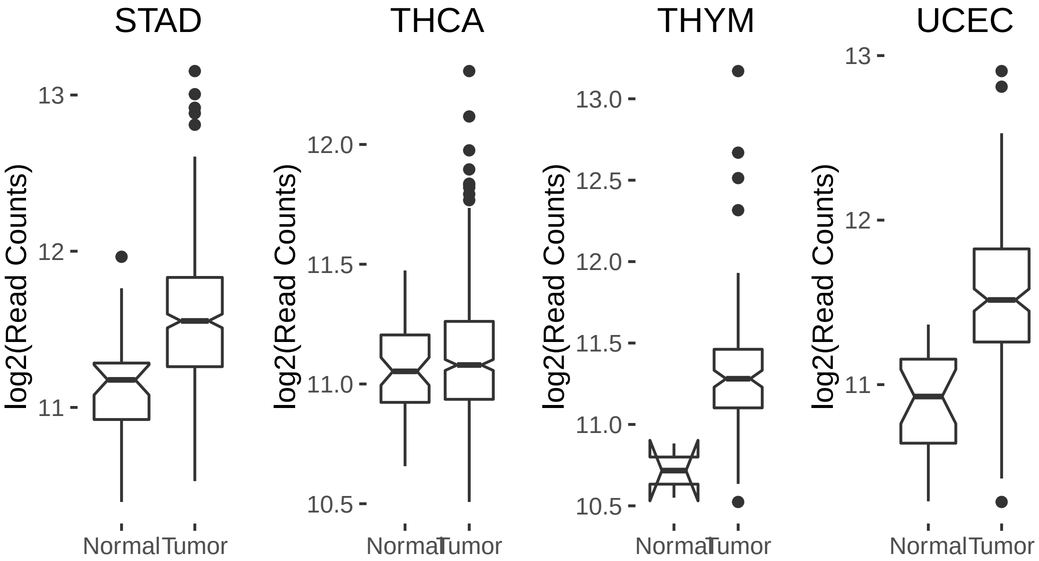
STAD
THCA
THYM
UCEC
log2(Read Counts)
13
12
11
12.0
11.5
11.0
10.5
13.0
12.5
Normal
Tumor
