# Supplementary figures and images for: The Mutational Landscape of the Oncogenic MZF1 SCAN Domain in Cancer
Source: Front Mol Biosci. 2016 Dec 15;3:78. doi: 10.3389/fmolb.2016.00078 (PMC5156680; doi:10.3389/fmolb.2016.00078)

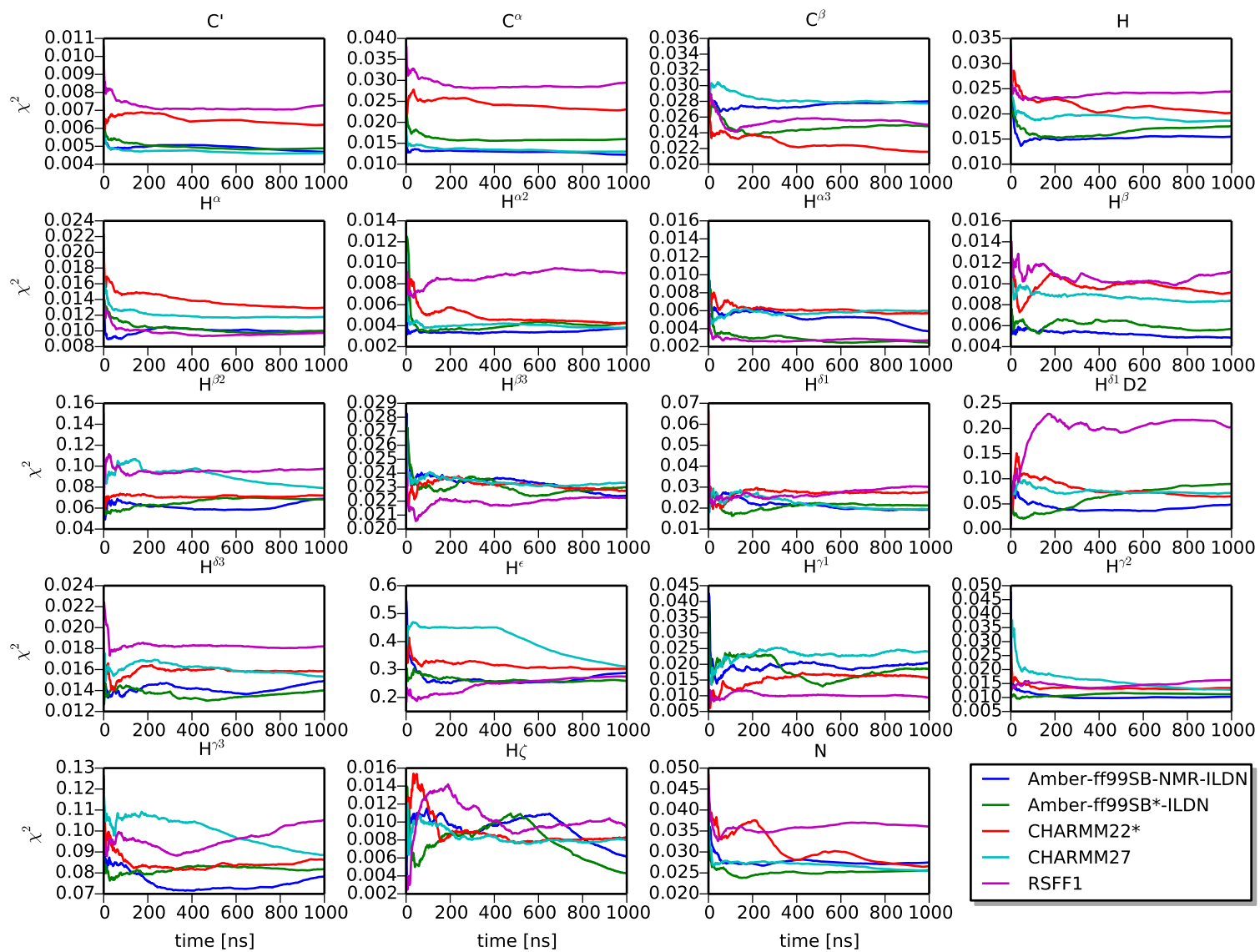

Supplement: Supplementary file 5 [file Image2.PDF]

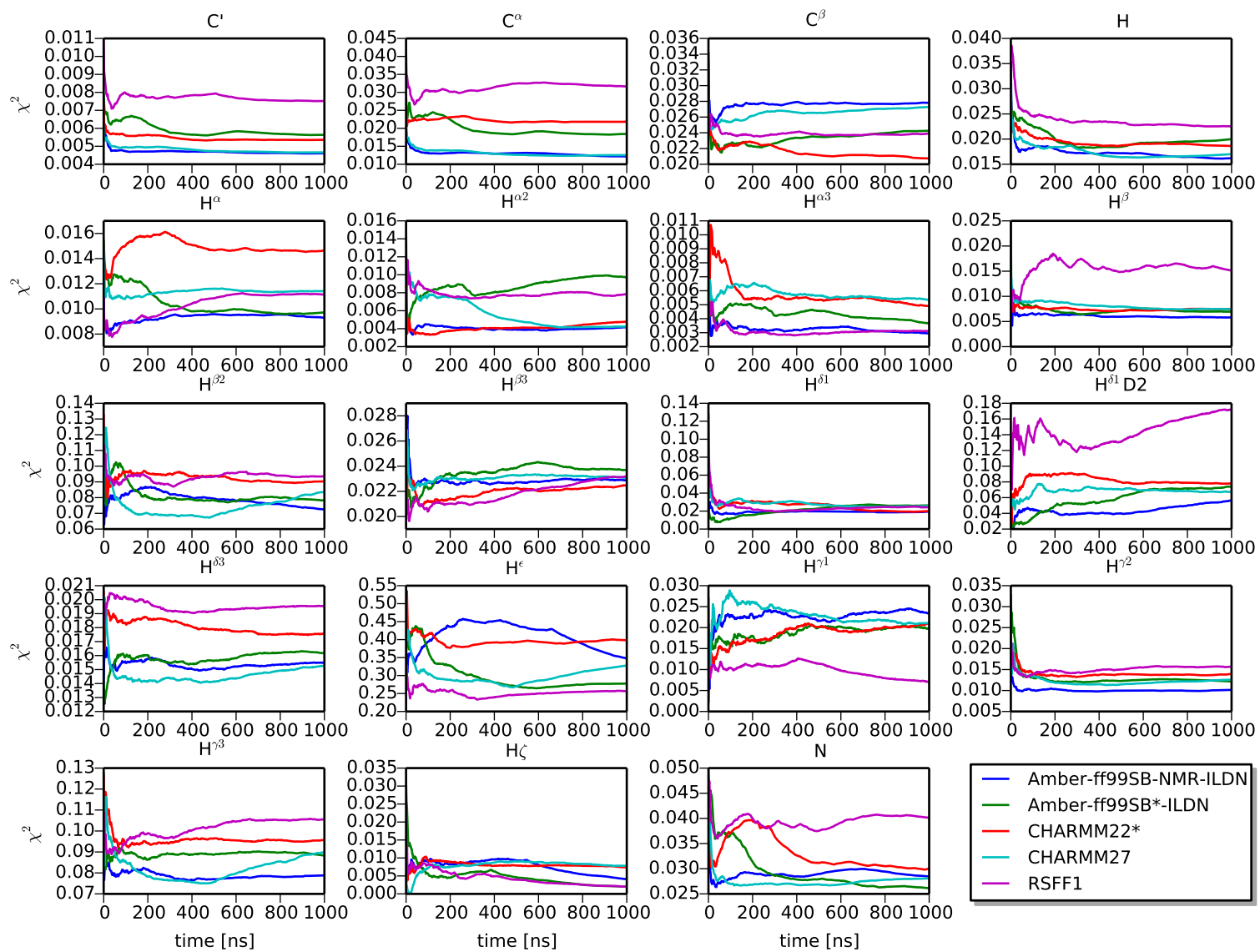

Supplement: Supplementary file 6 [file Image3.PDF]

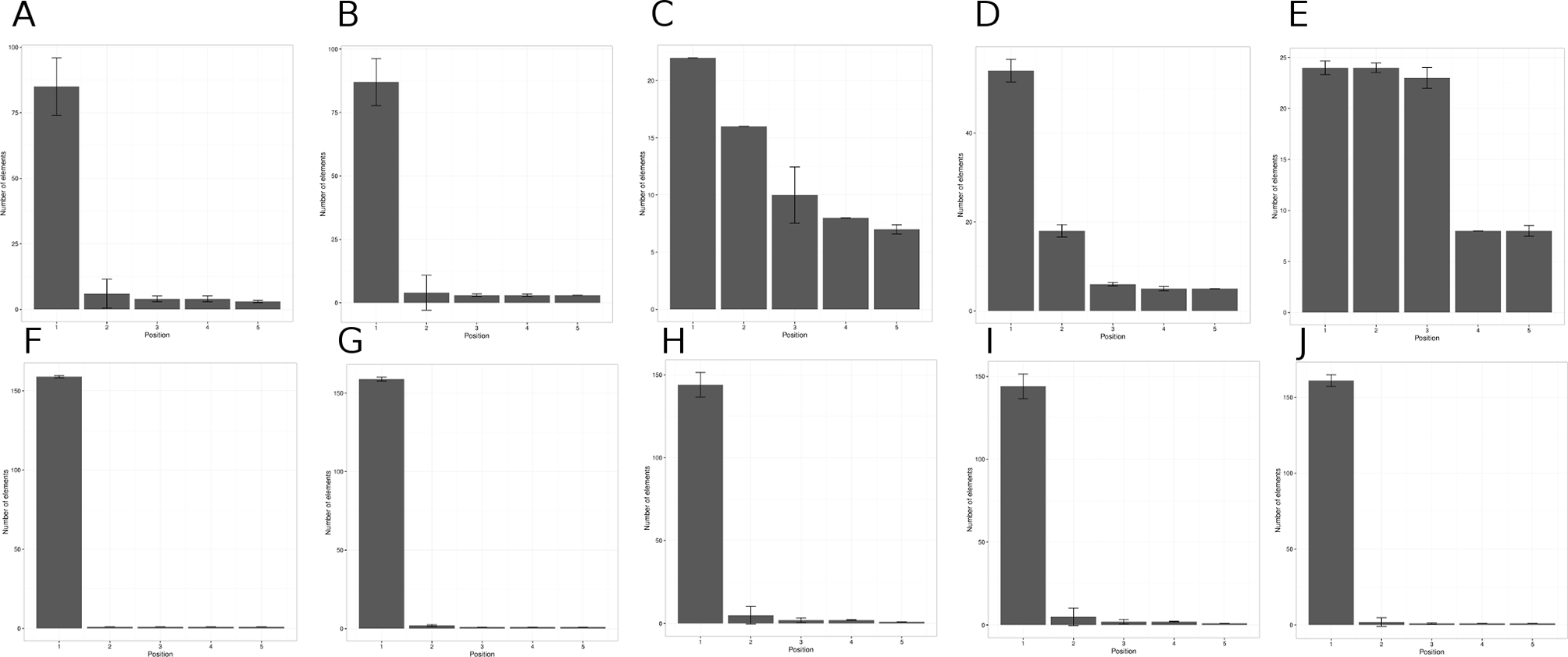

Supplement: Supplementary file 7 [file Image4.TIFF]

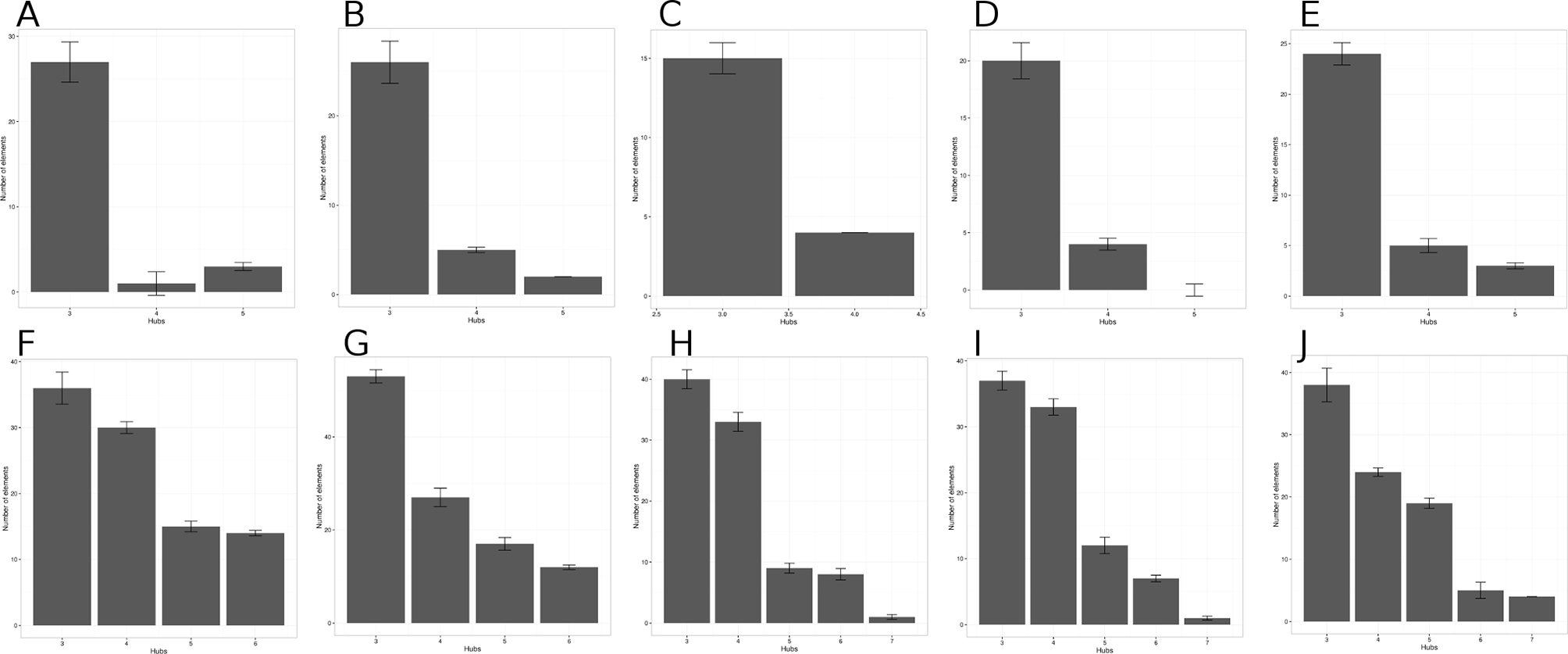

Supplement: Supplementary file 8 [file Image5.TIFF]
